# Model-based analysis of influenza A virus replication in genetically engineered cell lines elucidates the impact of host cell factors on key kinetic parameters of virus growth

**DOI:** 10.1371/journal.pcbi.1006944

**Published:** 2019-04-11

**Authors:** Tanja Laske, Mandy Bachmann, Melanie Dostert, Alexander Karlas, Dagmar Wirth, Timo Frensing, Thomas F. Meyer, Hansjörg Hauser, Udo Reichl

**Affiliations:** 1 Department of Bioprocess Engineering, Max Planck Institute for Dynamics of Complex Technical Systems, Magdeburg, Germany; 2 Department of Molecular Biology, Max Planck Institute for Infection Biology, Berlin, Germany; 3 Research Group Model Systems for Infection and Immunity, Helmholtz Center for Infection Research, Braunschweig, Germany; 4 Division of Experimental Hematology, Medical University Hannover, Hannover, Germany; 5 Department of Gene Regulation and Differentiation, Helmholtz Center for Infection Research, Braunschweig, Germany; 6 Chair of Bioprocess Engineering, Faculty of Process and Systems Engineering, Otto von Guericke University, Magdeburg, Germany; University of California, Los Angeles, UNITED STATES

## Abstract

The best measure to limit spread of contagious diseases caused by influenza A viruses (IAVs) is annual vaccination. The growing global demand for low-cost vaccines requires the establishment of high-yield production processes. One possible option to address this challenge is the engineering of novel vaccine producer cell lines by manipulating gene expression of host cell factors relevant for virus replication. To support detailed characterization of engineered cell lines, we fitted an ordinary differential equation (ODE)-based model of intracellular IAV replication previously established by our group to experimental data obtained from infection studies in human A549 cells. Model predictions indicate that steps of viral RNA synthesis, their regulation and particle assembly and virus budding are promising targets for cell line engineering. The importance of these steps was confirmed in four of five single gene overexpression cell lines (SGOs) that showed small, but reproducible changes in early dynamics of RNA synthesis and virus release. Model-based analysis suggests, however, that overexpression of the selected host cell factors negatively influences specific RNA synthesis rates. Still, virus yield was rescued by an increase in the virus release rate. Based on parameter estimations obtained for SGOs, we predicted that there is a potential benefit associated with overexpressing multiple host cell genes in one cell line, which was validated experimentally. Overall, this model-based study on IAV replication in engineered cell lines provides a step forward in the dynamic and quantitative characterization of IAV-host cell interactions. Furthermore, it suggests targets for gene editing and indicates that overexpression of multiple host cell factors may be beneficial for the design of novel producer cell lines.

## Author summary

Influenza viruses depend on cellular functions at every step of their life cycle and a comprehensive picture of virus-host cell interactions is the key to understand influenza disease and establish antiviral therapies. Over the past decade, this was supported by numerous screening approaches, which identified cellular factors relevant for intracellular virus replication. Ideally, the identification of pro-viral targets should also support the generation of cell lines to optimize influenza virus replication in cell cultures. As a first approach towards this goal, we used a mathematical model to identify mechanisms of viral growth that would be most promising targets for host cell factor manipulation. Based on predictions, we expected a significant increase in virus production if RNA synthesis and virus assembly and virus budding were perturbed, which was partially confirmed by cell lines overexpressing single and multiple selected host cell factors. However, the cell-specific productivity of engineered cell lines was not improved significantly and, according to model-based analysis, this can be explained by adverse changes in kinetic parameters of intracellular replication steps. Finally, results indicate that screening approaches should focus on late time points post infection to identify targets for engineering of cell lines that support high-yield vaccine production processes.

## Introduction

Influenza A viruses (IAVs) are highly contagious respiratory pathogens that constitute a permanent threat to public health, causing three to five million cases of severe illness and up 650,000 deaths per year [1]. As obligate intracellular parasites, influenza viruses rely on host cellular functions at every step of their life cycle. Thus, to deepen the understanding of virus-host cell interactions is a key step to improve vaccine production and thereby efficiently counteract disease. During the past decade multiple RNAi screens, yeast-two-hybrid approaches and omics studies, allowed for systematic identification of cellular factors that are relevant for the IAV life cycle (recently reviewed by [2]). These factors are commonly grouped into pro- and antiviral factors, which can be used to design new therapeutic and preventive disease measures. So far, the focus of these investigations was mainly on novel antiviral treatment that targets host dependency factors instead of viral factors, which might help to avoid the emergence of viral escape mutants [3–6]. Regarding the design of cell lines for optimized virus production, however, host restriction factors, e.g. factors that belong to cellular antiviral defense mechanisms and which can be downregulated to increase the virus yield in vaccine manufacturing, are of key importance. In the case of poliovirus, for instance, the knockdown of host cell factors that inhibit virus replication in adherent Vero cells was reported to result in a ten-fold increase in virus titers [7]. This promising result, however, could not be reproduced in a recent follow-up study [8]. Another option, pursued in our study, is the overexpression of host dependency factors to facilitate virus replication and increase yields in cell culture-based IAV production. To this end, we chose the lung carcinoma cell line A549 as a model cell line that was previously used in two genome-wide RNAi screens for identification of antiviral targets [9,10] (for further review of relevant RNAi screens the reader is referred to [11]). In these studies, changes in virus replication were measured in cells with temporal modulation of gene expression and evaluated at single time points post infection (p.i.). To complement this approach, we investigate the dynamics of virus replication in cell lines stably overexpressing host cell genes over an extended period.

Since virus-host cell interactions display highly complex dynamics, mathematical modeling approaches are crucial to support the interpretation of time courses of viral components measured in experiments, e.g. intracellular viral RNA copy numbers. In addition, such models help to explain specific steps and outcomes of virus-host cell interaction, to study effects of changes in expression of viral or cellular components, or to make predictions about phenotypic changes after cell line engineering, i.e., inhibition of virus growth or increase in yield. We employed a model of the IAV life cycle that describes virus replication within a single infected adherent MDCK cell [12]. First, we re-calibrated this model to experimental data from infected A549 cells obtained in this study. Second, we predicted which steps of the virus life cycle are most sensitive with respect to cell-specific virus yield and therefore represent promising targets for cell line engineering. To validate model predictions, we integrated various experimental data sets from infection studies performed in A549 cell lines that we modified genetically to overexpress host cell factors previously identified by RNAi screening [9,13–15] and studies performed by other research groups [16–19]. Finally, the resulting parameter sets for IAV replication in single gene overexpression cell lines (referred to as SGOs), were used to predict the outcome of IAV infection in multiple gene overexpression cell lines (referred to as MGOs). While only one of five of the selected SGOs showed a higher virus yield compared to the parental A549 cell line, MGO simulations indicated that there is a potential for a significant increase in virus yield. However, this finding was confirmed only partially in experiments.

Overall, SGOs and MGOs that were established during this study showed an improvement in early release dynamics rather than the expected increase in total virus yield compared to their parental cell line. Using a single cell model of IAV replication, we elucidate this in greater detail and link the overexpression of host cell factors to changes in key parameters of virus growth, which has not been reported before.

## Results

### Mathematical model for intracellular influenza A virus replication in A549 cells

The model of IAV replication used in this study is identical to a previously published description of the intracellular life cycle of IAV [12]. In general, we assume that basic mechanisms of IAV replication are similar in different host cell lines, but that values for key parameters of virus growth have to be adapted for each host cell system. While the previous model [12] was calibrated against various experimental data, mostly acquired from infected MDCK cells [20,21], the re-calibration of the model used in this study was based on three sets of in-house experimental data from infected A549 cells (S1 Fig). The available measurements allowed to estimate the kinetic parameters for nuclear import of vRNPs *k*^*Imp*^, the synthesis of viral mRNA, cRNA and vRNA ($k_{M}^{Syn}$, $k_{C}^{Syn}$, $k_{V}^{Syn}$) as well as binding of matrix protein 1 (M1) $k_{M1}^{Bind}$ and the release of viral progeny *k*^*Rel*^. Statistical testing (Table 1) revealed that $k_{C}^{Syn}$ and $k_{M1}^{Bind}$ were not significantly different in A549 compared to MDCK cells [12]. However, $k_{V}^{Syn}$ was significantly increased and $k_{M}^{Syn}$ significantly reduced in A549 cells, respectively.

**Table 1:** Comparison of key parameters of IAV replication in adherent MDCK and A549 cells.

Rate constant	Description	Value MDCK cells [12]	Value A549 cells	Unit
*k*^*Imp*^	Nuclear vRNP import	6 ^n.a.^	0.296	*h*^−1^
$k_{V}^{Syn}$	vRNA synthesis	13.86 ***	100.93	*h*^−1^
$k_{C}^{Syn}$	cRNA synthesis	1.38 ^n.s.^	1.53	*h*^−1^
$k_{M}^{Syn}$	mRNA synthesis	2.5x10^5^ **	3.06x10^4^	*nt*⋅*h*^−1^
$k_{M1}^{Bind}$	Binding of M1 to nuclear vRNPs	1.39x10^-6^ ^n.s.^	1.82x10^-6^	*molecule*^−1^⋅*h*^−1^
*k*^*Rel*^	Virus release	3.70x10^-3^ ^n.a.^	1.10x10^-3^	*virions*⋅*molecule*^−1^⋅*h*^−1^

n.a.–not assessed (no bootstrap simulations in [12] available)

asterisks indicate significant differences of the parameter values with respect to the A549 cell line for a one-sided Gauss test with

** p ≤ 0.01 and

*** p ≤ 0.001

n.s.–not significant.

### Model-based identification of potential bottlenecks in influenza A virus replication

Two simplifying assumptions were made to simulate the influence of host cell factors on IAV replication. First, we considered that each step in the virus life cycle was dependent on one host cell factor and secondly, that a change in the expression level of this host cell factor would directly translate into a change of the corresponding kinetic parameter value in our mathematical model for IAV replication. For instance, if a host cell factor responsible for vRNA synthesis is overexpressed, vRNA replication is enhanced, resulting in a higher vRNA synthesis rate. Likewise, the downregulation of the same factor would result in a reduced vRNA synthesis rate.

**Fig 1:**
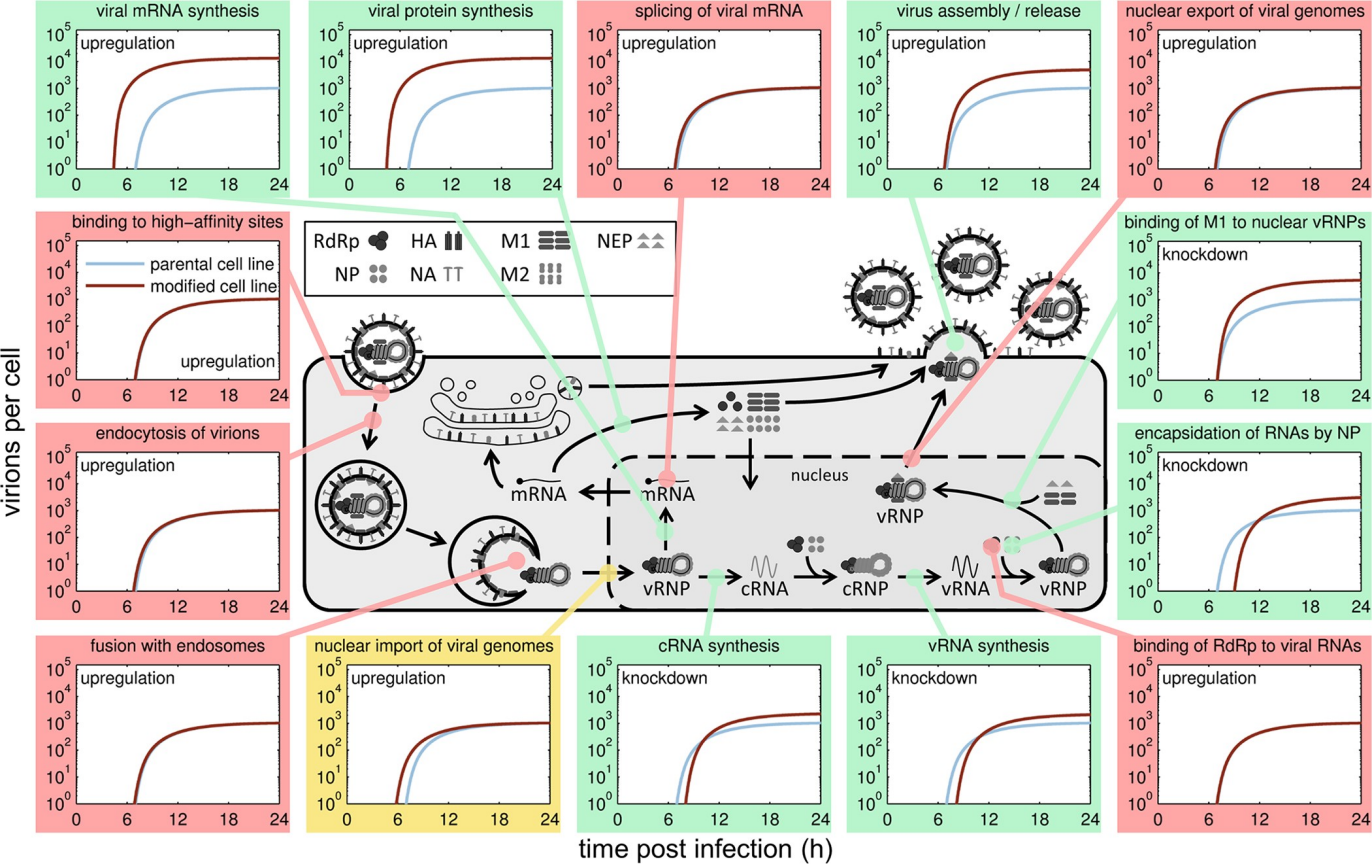
Virus release dynamics in response to *in silico* manipulation of gene expression of host cell factors in A549 cells. We assume that the efficiency of individual steps in the virus life cycle is directly dependent on host cell factors and their influence is changed upon knockdown or overexpression of the corresponding gene. We simulated manipulation of gene expression by perturbing the corresponding kinetic parameters of an IAV replication model for A549 cells, which is based on a model previously established by our group [12]. For a simulated infection at MOI 1, virus release of the parental A549 (blue solid line) and the engineered cell line (brown solid line) are shown for the most important virus replication steps. Colors indicate whether perturbation of the corresponding step improved virus yield at 24 h p.i. by at least two-fold (green), had only an impact on the starting time point of virus release (yellow) or no impact (red). Scheme of IAV replication adapted from [22].

**Fig 2:**
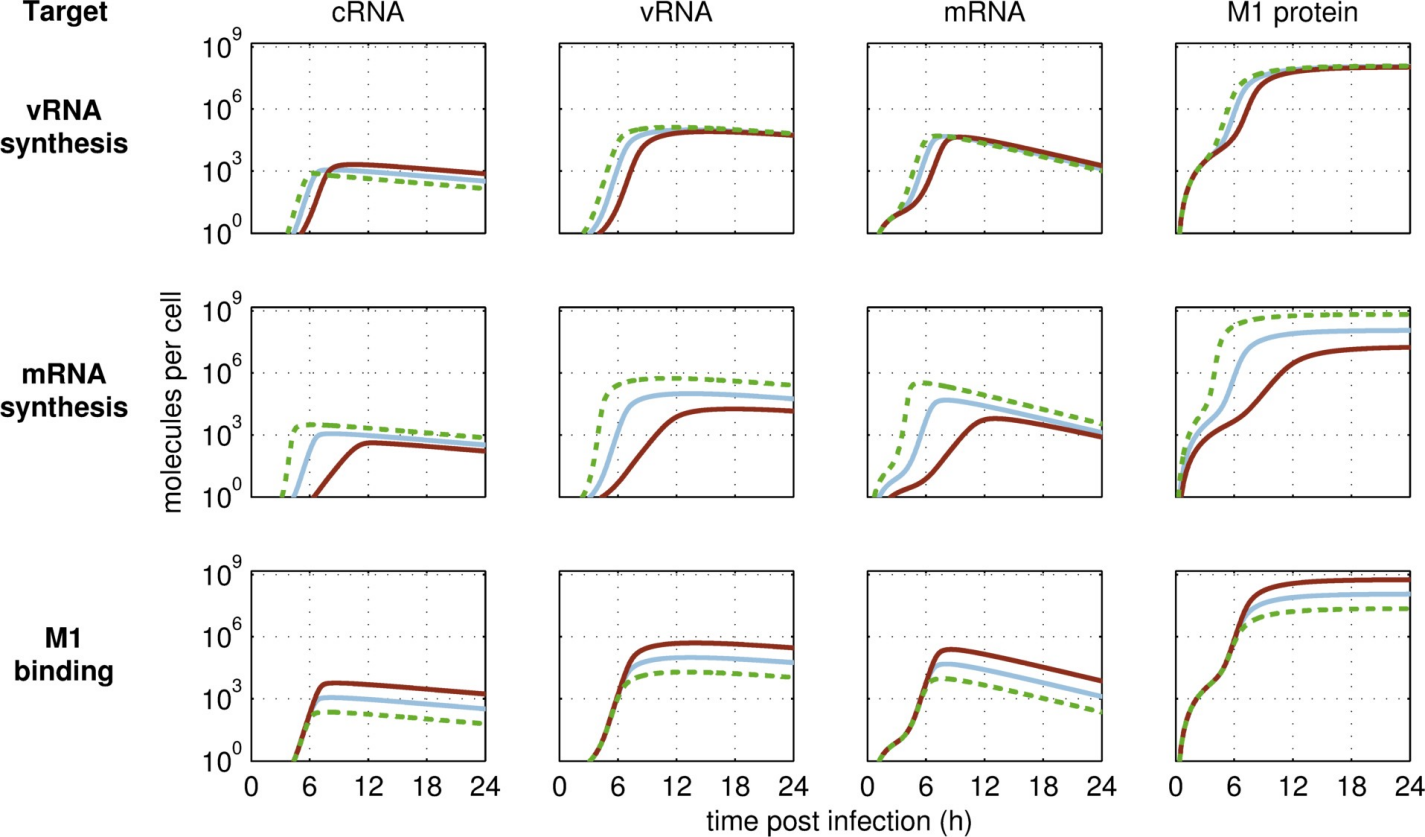
Intracellular replication dynamics in response to *in silico* modifications of host cell gene expression in a single infected cell. Changes in levels of viral cRNA (column 1), vRNA (column 2), mRNA (column 3) and matrix protein 1 (M1, column 4) are shown for a simulated infection at MOI 1 for the parental A549 cell line (blue solid line) or upon targeting selected steps of virus replication, as indicated on the left-hand side, by either knockdown (brown solid line) or overexpression (green dashed line).

Based on these assumptions, we performed *in silico* engineering of A549 cells by perturbing each parameter of our model individually with the objective to maximize virus yield at 24 h p.i. (optimized parameter values are summarized in S1 Table). By comparing the simulated virus release of parental A549 cells to results obtained for *in silico* optimized cell lines (Fig 1), we observed three possible outcomes upon parameter perturbation: (i) virus release dynamics were not affected significantly, (ii) only onset of virus release was improved, starting at least 1 h earlier compared to the parental A549 cell line and (iii) virus release dynamics were affected significantly leading to an increase in final yield by at least two-fold. The latter was caused by perturbations of parameters that define the most promising targets for cell line engineering, namely steps of viral RNA synthesis, its regulation and virus release (Fig 1, green shaded subfigures). Interestingly, the model predicted that the upregulation of viral mRNA synthesis is beneficial for virus replication whereas synthesis of viral cRNA and vRNA should be downregulated. To investigate this in greater detail we, next, compared the dynamics of the simulated intracellular viral RNAs and protein levels in both upregulation and downregulation scenarios to levels in parental A549 cells (Fig 2). We observed that changes of intracellular replication dynamics were most evident upon manipulation of viral mRNA synthesis (Fig 2, middle panel). Most importantly, the sole increase of the mRNA synthesis rate lead to a higher increase in vRNA levels than the upregulation of the vRNA synthesis rate itself (Fig 2, upper and middle panel second column). This strongly indicates that viral RNA replication in A549 cells is already saturated and only if more viral mRNA, and consequently, more viral proteins were available, more vRNA could be produced and virus release could be enhanced significantly. In addition, the modulation of regulatory steps, which is accounted for in our model by binding of M1 (negative regulator), had only an impact on final RNA and protein levels rather than on the dynamics *per se* (Fig 2, bottom panel).

### Screening of different cell lines based on HA titer upon infection at MOI 10^−4^

To validate our model predictions, we used lentiviral gene transfer to generate A549 cell populations that overexpress specific host cell genes relevant for IAV replication. The host cell factors CEACAM6, FANCG, NXF1, PLD2 and XAB2 were selected from a set of candidate genes determined previously by RNAi screening [9,13–15] and virus-host cell interaction studies [16,17]. An overview of genes and their function in the IAV life cycle is given in S2 Table. The resulting cell populations were subjected to fluorescence activated cell sorting (FACS) to enrich cells that express the transduced gene based on eGFP, which is the co-expressed reporter gene. SGOs that showed stable gene overexpression were infected with A/Puerto Rico/8/34 (A/PR/8/34, H1N1) at a multiplicity of infection (MOI) of 10^−4^, which is usually applied for vaccine production processes. We compared virus titers of each SGO to that of the parental A549 cell line at selected time points p.i. (Table 2). Assuming that changes in virus release are associated with changes in intracellular mechanisms, we selected SGOs for further characterization of intracellular virus replication based on their HA titer. To facilitate selection, we ranked the HA measurements for each time point and each cell line according to their relative increase compared to the parental A549 cell line. As can be seen by the measurement data and the corresponding ranking values in Table 2, HA titers of all SGOs were increased at early time points p.i., whereas none of the SGOs showed an increase greater than 20% of the final HA titer at the usual time of harvest 72 h p.i. Thus, by modulating the expression level of these host cell factors, it was possible to influence the IAV release dynamics, however, the total virus yield was similar comparing SGOs to their parental cell line.

**Table 2:** HA titers and ranking results of cell lines overexpressing single host cell genes infected with A/PR/8/34 (H1N1) at MOI 10^−4^.

	HA titer^+^ (log_10_ HA units/100 μL)				Ranking value^#^ (-)			
time p.i. (h)	36	42	72	96	36	42	72	96
**A549**	1.05	1.44	1.85	1.97	0	0	0	0
**control**	0.97	1.39	1.60	1.87	0	0	0	0
**CEACAM6**	1.13	1.40	1.80	1.78	1	0	0	0
**FANCG**	1.27	1.55	1.76	1.85	3	1	0	0
**NXF1**	1.37	1.65	1.90	1.91	5	3	0	0
**PLD2**	1.16	1.49	1.91	1.88	1	0	0	0
**XAB2**	1.22	1.48	1.73	1.79	2	0	0	0

^+^ Evaluation of HA titers by color shading indicates the higher the value the darker the shading

^#^ Zero, 1, 2, 3, 4, 5 for increase in log HA titer by < 20%, ≥ 20%, ≥ 40%, ≥ 60%, ≥ 80%, ≥ 100%, respectively

See S3 Table for the level of overexpression of the corresponding genes.

### Detailed experimental characterization and determination of kinetic parameters for cell lines overexpressing selected host cell genes

**Fig 3:**
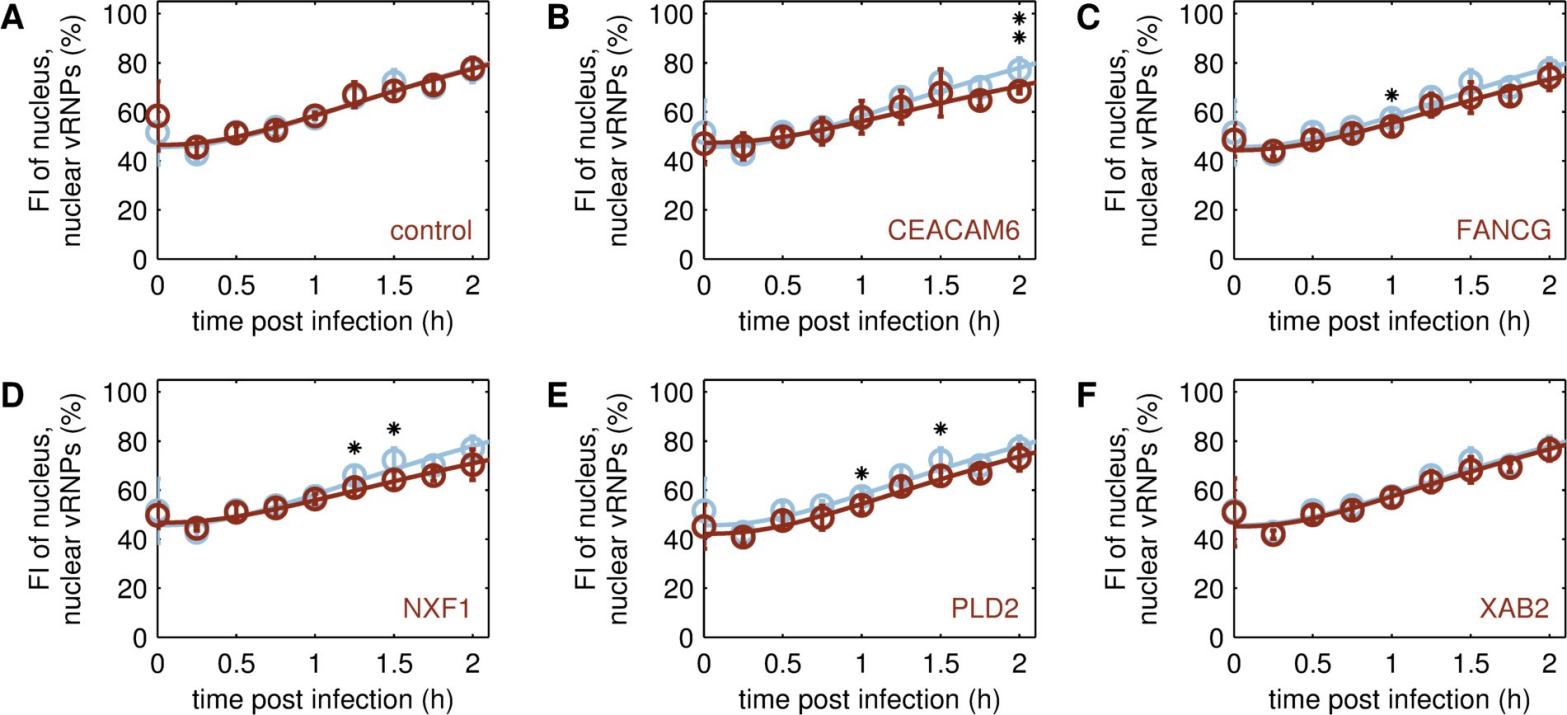
Nuclear import of viral genomes in different A549 cell lines. Model fit (lines) to experimental data (circles ± standard deviation, n = 4) for the import of viral genomes (vRNPs) in cycloheximide-treated cell lines upon infection by A/PR/8/34 (H1N1) at MOI 50. Relative increase in fluorescence intensity (FI) of the nucleus was determined by imaging flow cytometry after co-staining of cells with DAPI and vRNP antibody. The nuclear import rate was estimated by fitting the simulated fraction of nuclear vRNPs to the averaged experimental data. To account for the background signal of the nucleus in images, an offset of approximately 50% at 0 h p.i. was applied with respect to the experimental data obtained for parental A549 cells (A-F, blue), the transduction control (A, brown) and engineered cell lines overexpressing one of the following host cell factors: CEACAM6 (B, brown), FANCG (C, brown), NXF1 (D, brown), PLD2 (E, brown), XAB2 (F, brown). The asterisks indicate differences in relative FI levels with respect to the parental A549 cell line, that were either noticeable, however, statistically not significant (* p ≤ 0.1), or significant (** p ≤ 0.05) determined by Kruskal-Wallis test.

**Fig 4:**
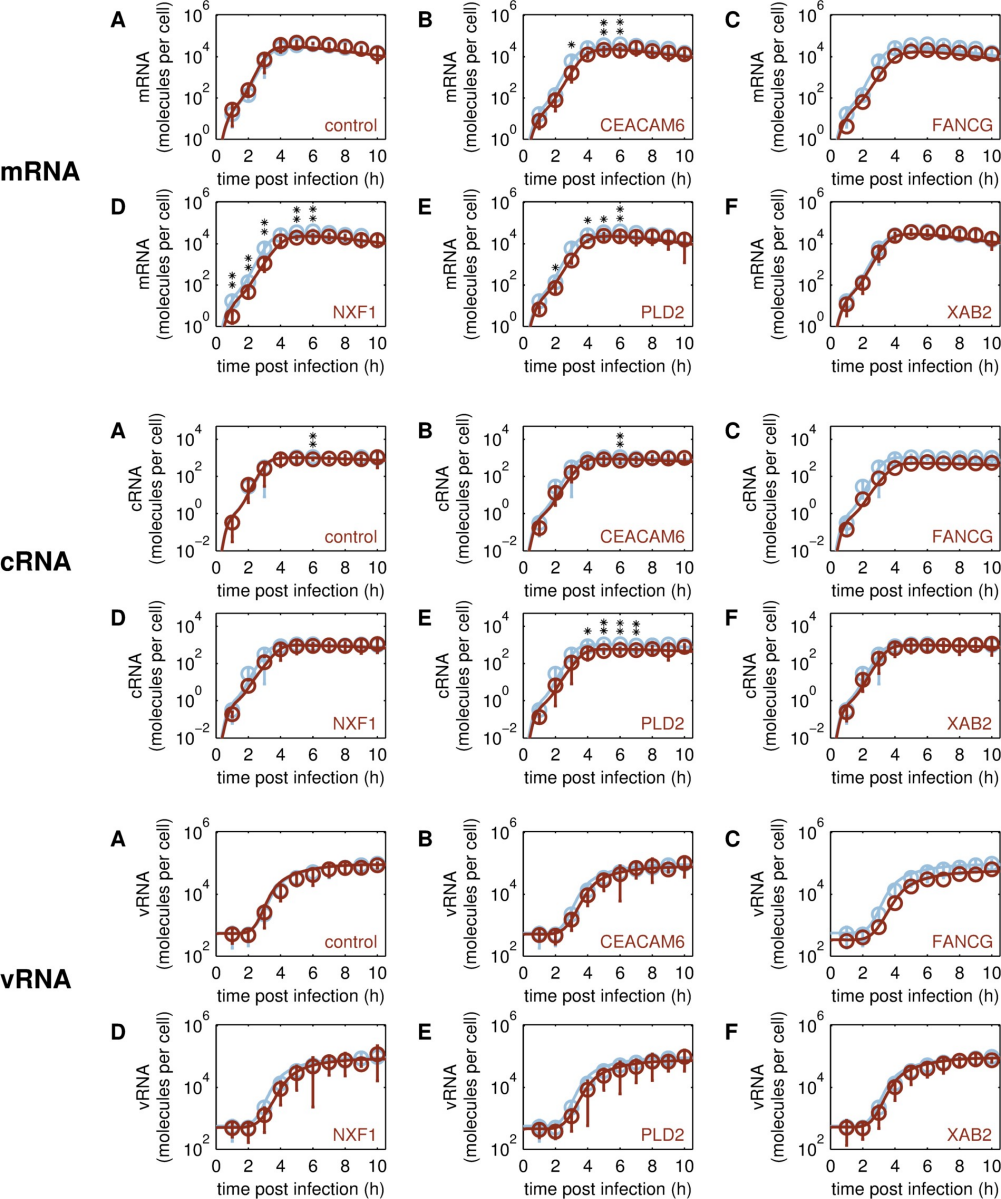
Intracellular dynamics of viral RNA synthesis in different A549 cell lines. Model fit (lines) to experimental data (circles ± standard deviation, n = 4 or single circles for FANCG, n = 2) of viral mRNA (panel 1), cRNA (panel 2), vRNA (panel 3) of segment 5 (encoding NP) in cell lines infected with A/PR/8/34 (H1N1) at MOI 50. Viral RNA synthesis rates and M1 binding rate were estimated by fitting the simulated number of the three viral RNA species to averaged segment-specific RT-qPCR data. To account for the offset in vRNA measurements caused by free viral RNAs in the seed virus we also implemented such offsets in our simulations with respect to the measurements obtained for parental A549 cells (A-F, blue), the transduction control (A, brown) and engineered cell lines overexpressing one of the following host cell factors: CEACAM6 (B, brown), FANCG (C, brown), NXF1 (D, brown), PLD2 (E, brown), XAB2 (F, brown). The asterisks indicate differences in RNA levels with respect to the parental A549 cell line, that were either noticeable, however, statistically not significant (* p ≤ 0.1), or significant (** p ≤ 0.05) determined by Kruskal-Wallis test.

**Fig 5:**
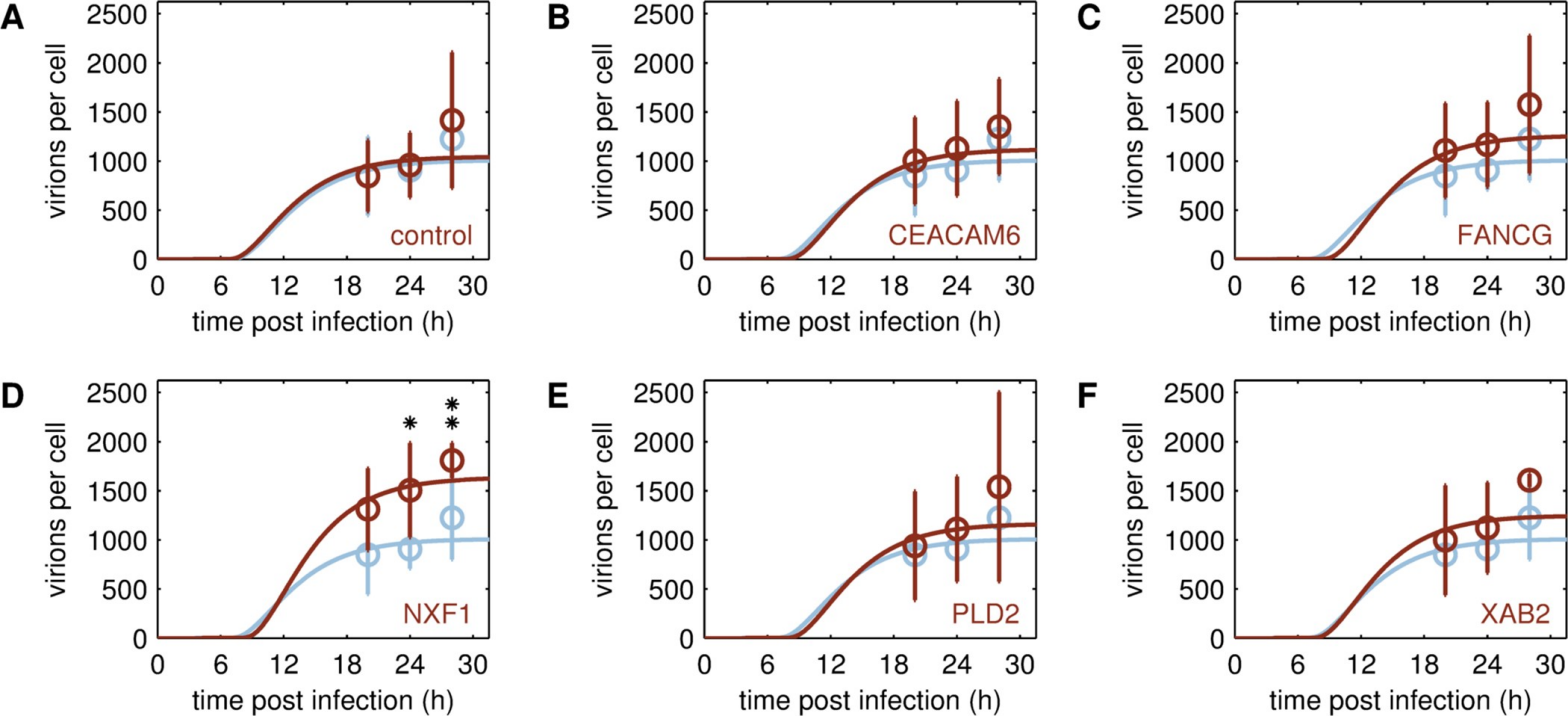
Virus particle release of different A549 cell lines. Model fit (lines) to cell-specific numbers of released virions estimated from HA titer and maximum viable cell count (circles ± standard deviation, n ≥ 4) obtained from A/PR/8/34 (H1N1) infections at MOI 1. Simulated number of released virions was fitted to averaged cell-specific yield obtained for parental A549 cells (A-F, blue), the transduction control (A, brown) and engineered cell lines overexpressing one of the following host cell factors: CEACAM6 (B, brown), FANCG (C, brown), NXF1 (D, brown), PLD2 (E, brown), XAB2 (F, brown). The asterisks indicate differences in cell-specific yield with respect to the parental A549 cell line, that were either noticeable, however, statistically not significant (* p ≤ 0.1), or significant (** p ≤ 0.05) determined by Kruskal-Wallis test.

Next, we performed a detailed characterization of intracellular steps of viral growth in IAV-infected SGOs as well as in the parental A549 cells and an eGFP transduction control (Figs 3–5).

#### Nuclear import of viral genomes

We examined nuclear import of vRNPs in A549 cells with the help of imaging flow cytometry, which combines both the statistically relevant throughput of cell counts known from conventional flow cytometry and the information on localization of the fluorescence signal inside a single cell usually acquired by fluorescence microscopy. Cells were treated with cycloheximide (CHX) to inhibit translation, such that only incoming vRNPs, resulting from virus uptake, would be detected in infected cells co-stained with DAPI and an anti-vRNP antibody.

**Fig 6:**
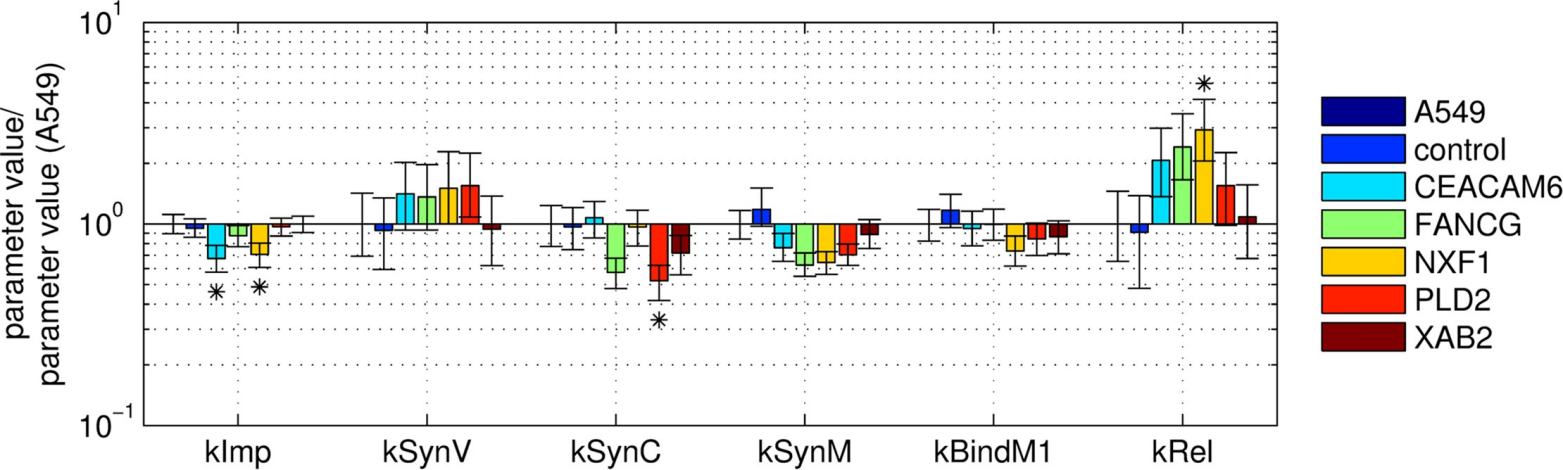
Comparison of parameter values for viral kinetics obtained for A/PR/8/34 (H1N1) infections in different A459 cell lines. After fitting 3000 resamplings of the available experimental data, all parameter values were normalized to the median of each kinetic parameter obtained for parental A549 cells. Bars represent the normalized medians and error bars indicate the first and third normalized quartile of each parameter per cell line (detailed boxplots in S2 Fig). The asterisk indicates differences in parameter values with respect to the parental A549 cell line, that were noticeable, however, statistically not significant (p ≤ 0.1) as calculated by a one-sided Z test (Gauss test).

Overall, the kinetics of nuclear vRNP import were similar in all tested cell lines (Fig 3). In particular, the transduction control (Fig 3A) and the XAB2 SGO (Fig 3F) showed exactly the same time course of nuclear vRNP import as the parental A549 cell line. FANCG and PLD2 SGOs showed slightly reduced levels (Fig 3C and 3E), while CEACAM6 and NXF1 showed a slightly slower increase of relative nuclear fluorescence intensity over time (Fig 3B and 3D). Using the mathematical single cell model, we estimated the nuclear import rate of viral genomes *k*^*Imp*^ for each cell line. While the differences in parameter values were statistically not significant with respect to the parental A549 cell line, we observed a trend showing a slight reduction (p ≤ 0.1, calculated by one-sided Gauss test) of *k*^*Imp*^ for CEACAM6 and NXF1 SGOs (Fig 6).

#### Viral replication and transcription

Next, we analyzed the intracellular replication and transcription dynamics of IAV RNA by segment-specific RT-qPCR. Therefore, we infected A549 cells at MOI 50 and measured viral mRNA, cRNA and vRNA of segment 5, which encodes the viral nucleoprotein (NP).

Overall, the dynamics of the three viral RNA species were similar in all five SGOs compared to the parental A549 cell line (Fig 4). A few trends (statistically not significant) were found in intracellular RNA measurements. In particular, viral mRNA levels in CEACAM6 (Fig 4, upper panel, B), NXF1 (Fig 4, upper panel, D), and PLD2 SGOs (Fig 4, upper panel, E) seemed to be reduced at time points ≤ 6 h p.i. Interestingly, viral cRNA levels were reduced significantly (p ≤ 0.05) in the PLD2 SGO from 4 to 7 h p.i. (Fig 4, middle panel, E), while no significant differences in cRNA levels were evident for other SGOs. Although we observed a slight reduction of mRNA and/or cRNA in some of the infected SGOs, the time courses of vRNA synthesis and the number of viral genome copies per cell were similar for most tested cell lines (Fig 4, bottom panel). Only the FANCG SGO is an exception, since vRNA levels in infected FANCG SGO cells were reduced as measured in two independent experiments. Furthermore, viral mRNA and cRNA levels were also reduced in FANCG SGO cells. According to the Kruskal-Wallis test, the difference in the raw data compared to those of parental A549 cells was statistically not significant. However, statistical testing could be performed on the empiric parameter distributions, generated upon multiple resampling of the intracellular viral RNA measurement data and repeated model fitting (Fig 6). For this, we fitted the time courses of the three RNA species simultaneously to estimate the synthesis rates of mRNA $k_{M}^{Syn}$, vRNA $k_{V}^{Syn}$, cRNA $k_{C}^{Syn}$ and the binding rate of the negative regulator M1 to vRNPs $k_{M1}^{Bind}$. In agreement with the experimental data, model-based analysis revealed that the mRNA synthesis rate $k_{M}^{Syn}$ and cRNA synthesis rate $k_{C}^{Syn}$ were reduced in most of the SGOs (Fig 6). In particular, both $k_{M}^{Syn}$ and $k_{C}^{Syn}$, were also reduced for the FANCG SGO. Furthermore, the synthesis rate of vRNA $k_{V}^{Syn}$ was estimated to be slightly higher in SGOs compared to the parental A549 cell line. Since all three viral RNA species engage in an autocatalytic cycle, the synthesis rate of vRNA $k_{V}^{Syn}$ has to be increased in order to maintain vRNA levels comparable to the parental A549 cell line and therefore compensates for reduction of either $k_{C}^{Syn}$ or $k_{M}^{Syn}$ in infected SGOs. However, the increase of $k_{V}^{Syn}$ was not significant for any of the SGOs. Similarly, model-based analysis of the M1 binding rate $k_{M1}^{Bind}$ in infected SGOs revealed no significant changes compared to the parental A549 cell line.

#### Virus release

In addition to intracellular viral RNA levels, we also integrated experimental data of total virus release based on HA titer into our model to estimate the virus release rate *k*^*Rel*^ of SGOs (Fig 5). In contrast to our screening experiment for which cells were infected at MOI 10^−4^, we had to apply a higher MOI for model fitting. This was necessary since our single cell model cannot describe the progression of infections with multiple cycles in a cell population, which occur in low MOI scenarios. Therefore, we infected cells at MOI 1 and estimated the cell-specific virus release rate *k*^*Rel*^ with respect to the experimental data. Contrary to our expectations, the differences in the number of released virions were even less pronounced in this experiment compared to the initial cell line screening (Table 2). Only the NXF1 SGO showed significant differences in the number of released virions compared to the parental cell line (Fig 5D), which is also in line with a noticeable, although not significant, increase of the virus release rate *k*^*Rel*^ compared to the parental A549 cell line (Fig 6). Interestingly, also other SGOs showed an increase of *k*^*Rel*^ of about two-fold. This can be explained by the model’s architecture that leads to a compensation of the adverse/disadvantageous parametrization of viral replication and transcription through an increase in *k*^*Rel*^, which finally allows the model to capture the cell-specific virus yield determined in experiments.

### Computational investigation of cell lines overexpressing multiple host cell genes

**Fig 7:**
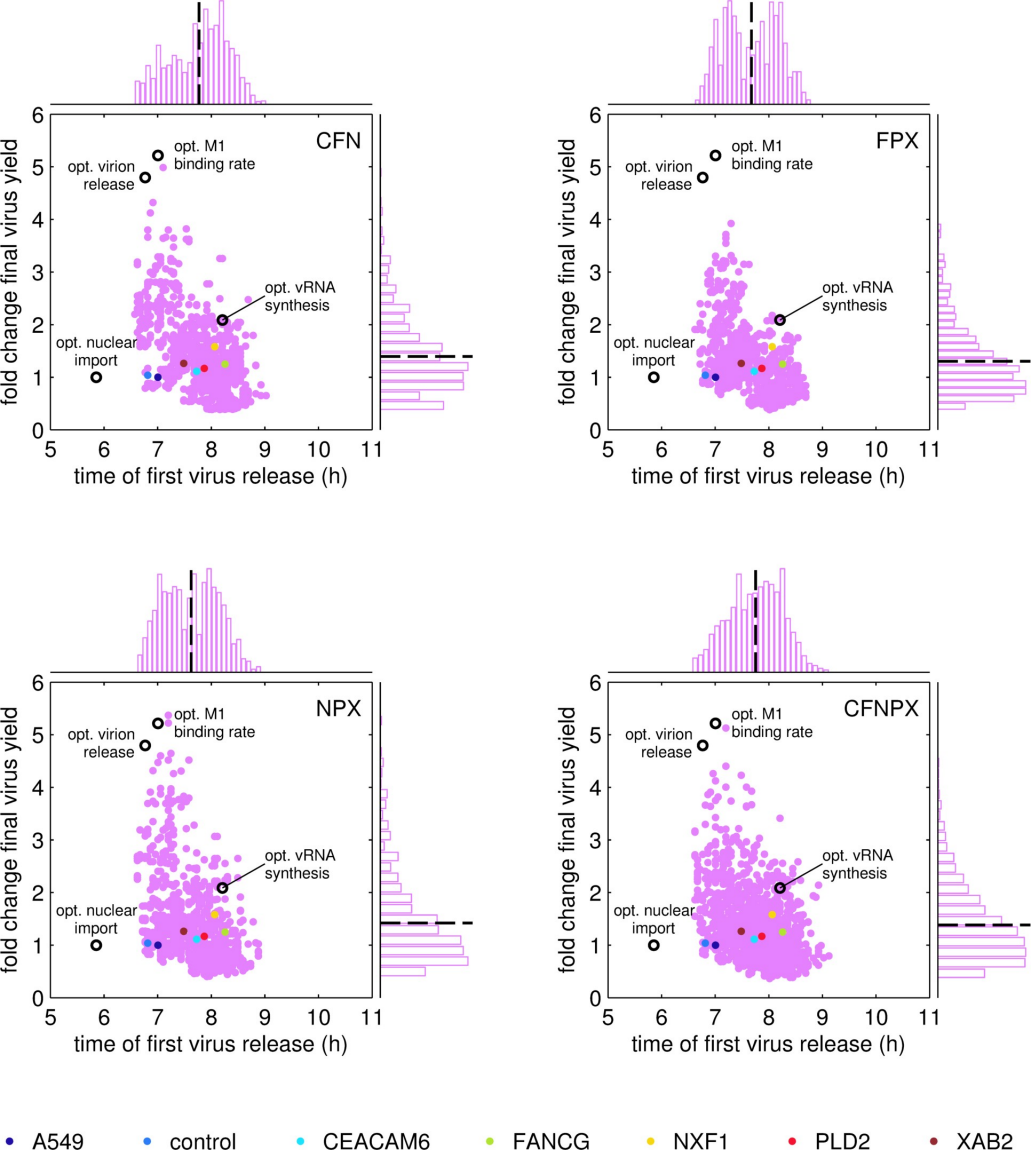
Evaluation of the time point of first virus release and the fold change in virus yield for model predictions of cell lines overexpressing multiple and single host cell genes. Multiple gene overexpression cell lines (MGOs) were generated *in silico* by random assembly of kinetic parameter sets based on experimental single gene overexpression cell lines (SGOs), where letters in the upper right corner indicate which gene combinations were simulated (genes names are abbreviated as their first letter). For the resulting MGOs (pink dots) every 10th of approximately 1 x 10^4^ - 2 x 10^4^ model predictions are shown and compared to simulations with parameter sets experimentally determined for SGOs and parental A549 cells (dots, colors according to legend) at 48 h p.i. for a simulated infection at MOI 1, cell-specific virus yields were normalized to the one obtained for parental A549 cells. Open circles represent single cell predictions using the indicated optimal parameter according to the analysis shown in Fig 1. Dashed lines in histograms indicate the arithmetic mean of the corresponding simulation readout.

Although only NXF1 SGOs showed a promising increase in virus yield, it seemed that overexpression of host cell factors can influence IAV replication on the intracellular level. Thus, we also explored the possibility whether additive or even synergistic effects on IAV yield could be achieved by overexpressing multiple host cell factors simultaneously. At first, we investigated this option by a computational approach and simulated the virus release of single cells overexpressing different combinations of multiple host cell factors. Since integration of genes into the host chromosome is random, the gene constructs will be inserted at different chromosomal locations with different transcriptional activities and, since transduction follows a Poisson distribution, not every cell will obtain the same number of the gene constructs. Together, these factors influence the strength of overexpression. In addition, the integration process can also have an impact on the gene expression through off-target effects. To account for all these scenarios, which involve some sort of randomness, we used randomized sets of parameters assembled based on the median values of the model parameters *k*^*Imp*^, $k_{V}^{Syn}$, $k_{C}^{Syn}$, $k_{M}^{Syn}$, $k_{M1}^{Bind}$ and *k*^*Rel*^, previously estimated from experimental data of infected SGOs and the parental A549 cell line. The parameter set of the latter was also included to account for off-target effects. For instance, the parameter set of an MGO may be composed of *k*^*Imp*^ of XAB2 SGOs, $k_{V}^{Syn}$ of PLD2 SGOs, $k_{C}^{Syn}$ of NXF1 SGOs, $k_{M}^{Syn}$ of FANCG SGOs, $k_{M1}^{Bind}$ of CEACAM6 SGOs, and *k*^*Rel*^ of the parental A549 cell line. We assume that all transduced genes can be expressed theoretically with the same probability, i.e., that there is an equal chance that kinetic parameters of the SGOs will be selected during randomization. Note, that even if all five candidate genes were transduced, not every MGO single cell will be a phenotypic mixture of all SGOs, but its parameter set could be *k*^*Imp*^ and $k_{V}^{Syn}$ of the parental A549 cell line, $k_{C}^{Syn}$ and $k_{M}^{Syn}$ of CEACAM6 SGOs and $k_{M1}^{Bind}$ and *k*^*Rel*^ of the NXF1 SGOs. To generate *in silico* MGOs, we chose to randomize parameter sets of those SGOs that showed a beneficial change in parameters according to initial model predictions of this study (Fig 1). Thus, we combined parameter sets of the top three candidates with the highest virus release rate *k*^*Rel*^ (CEACAM6 (C), FANCG (F) and NXF1 (N), CFN in Fig 7), the top three with the lowest cRNA synthesis rate $k_{C}^{Syn}$ (FANCG (F), PLD2 (P) and XAB2 (X), FPX in Fig 7), and the top three with the lowest M1 binding rate $k_{M1}^{Bind}$ (NXF1 (N), PLD2 (P), XAB2 (X), NPX in Fig 7). Finally, we also randomized parameter sets of all SGOs (CFNPX in Fig 7).

In a Monte Carlo approach, we generated multiple randomized parameter sets according to the selected combinations of SGOs and simulated virus infection at MOI 1 for 48 h (S3 Fig). Finally, we evaluated every single cell simulation for the time point at which the first simulated virus particle was released *t*(*V*^*Rel*^≥1) and the fold change in the maximum number of released viral progeny (Fig 7). Interestingly, these model predictions revealed that a single cell overexpressing multiple genes can theoretically yield up to five-fold more virus progeny than its parental cell line if the underlying parameter set was *k*^*Imp*^ and $k_{M}^{Syn}$ of the parental A549 cell line, $k_{V}^{Syn}$ of XAB2 SGOs, $k_{C}^{Syn}$ of PLD2 SGOs, and $k_{M1}^{Bind}$ and *k*^*Rel*^ of the NXF1 SGOs. In particular, the earlier virus release started, the higher was the fold increase in the number of viral progeny. While the time point of first virus release followed a normal distribution, the fold change of virus release showed a log-normal distribution with highly productive cells as rare events. Overall, the combinations CFN, NPX and CFNPX showed similar distributions of the simulation read outs, whereas the combination of FPX resulted in a narrower distribution of virus yield with a slightly lower maximum fold increase of four-fold. Finally, this analysis revealed that highly productive cells are rare events in a heterogenous MGO population and their contribution to the population average is negligible, which leads to an increase of less than two-fold in the final virus yield (Fig 7, dashed line in vertical histograms).

### Experimental evaluation of cell lines overexpressing multiple host cell genes

The computational analysis of MGOs indicated that overexpressing multiple host cell factors could result in an earlier onset of virus release and, to some extent, also in an improvement of virus yield. To validate these model predictions, we generated populations of A549 cells in which individual cells express random combinations of selected host cell factors at various levels (S4 Table). In particular, we generated three independent cell populations (MGO 1-3) which provide random combinations of all five host cell factors CFNPX, which also covers the phenotypes of combinations CFN and NPX according to simulations. Further, we generated MGO 4 in which the three factors FPX were randomly combined and which should show a slightly different phenotype compared to CFNPX. All MGOs were infected with IAV at MOI 10^-4^. We chose this MOI according to the SGO screening experiment (Table 2) since under these experimental conditions differences between cell lines were more pronounced than for infections at MOI 1 (Fig 5). Ranking of HA titers revealed that virus release of MGOs was increased at early time points, while final virus yield was not increased significantly in these cell populations compared to the parental A549 cell line (Table 3). Of note, the impact of overexpressing single host cell genes on virus yield could be enhanced by overexpressing multiple of these host cell genes simultaneously, which partially confirms our model predictions on MGOs. In addition, MGO 4 was the only cell line showing less than 40% increase in virus yield at 42 h p.i. compared to the parental A549 cell line. This supports the model prediction that the combination FPX results in a slightly less productive phenotype than other gene combinations.

**Table 3:** HA titers and ranking results of cell lines overexpressing multiple host cell genes infected with A/PR/8/34 (H1N1) at MOI 10^−4^.

	HA titer^+^ (log_10_ HA units/100 μL)				Ranking value^#^ (-)			
time p.i. (h)	36	42	72	96	36	42	72	96
**A549**	1.05	1.44	1.85	1.97	0	0	0	0
**MGO 1**	1.49	1.67	1.96	1.92	5	3	0	0
**MGO 2**	1.56	1.69	2.01	2.02	5	3	2	0
**MGO 3**	1.55	1.67	2.01	1.95	5	3	2	0
**MGO 4**	1.38	1.56	1.89	1.79	5	1	0	0

^+^ Evaluation of HA titers by color shading indicates the higher the value the darker the shading

^#^ Zero, 1, 2, 3, 4, 5 for increase in log HA titer by < 20%, ≥ 20%, ≥ 40%, ≥ 60%, ≥ 80%, ≥ 100%, respectively

MGO 1–3 overexpress all five host cell genes, MGO 4 overexpresses three host cell genes (FANCG, PLD2, XAB2)

See S4 Table for the level of overexpression for the corresponding genes.

## Discussion

### Influenza A virus replication in A549 cells

IAVs depend on host cellular functions to complete their replication cycle. Our aim was to take advantage of this dependency and manipulate the expression of host cell factors that are relevant for IAV replication to improve virus production for vaccine manufacturing. Due to the complexity of virus-host cell interactions mathematical models are required to complement the interpretation of infection experiments. In the present study, we used a re-calibrated model of IAV replication to predict and quantify changes in virus replication in genetically engineered A549 cells.

To account for the influence of host cell factors on steps of the virus life cycle, we made the simplifying assumption that changes in host cell gene expression have a direct impact on kinetic parameters of our model. Although we did not explicitly model physical interactions between host cell factors or cellular pathways with viral components, we were able to identify targets for cell line engineering by evaluating changes in the cell-specific virus release upon parameter perturbations. According to this *in silico* analysis, both a significant increase in virus yield as well as an earlier onset of virus release could be expected if either viral transcription or translation were significantly enhanced. In contrast, the model predicted that various steps of virus replication need to be downregulated to achieve a higher cell-specific virus yield. For instance, the binding of M1 to nuclear vRNPs, which mediates the nuclear export of vRNPs, should be delayed. The lower the binding rate of M1 $k_{M1}^{Bind}$, the longer vRNPs serve as template for viral genome replication and transcription inside the nucleus. Accordingly, not only more viral genome copies but also mRNAs will be synthesized and, thus, higher viral protein levels will be achieved (Fig 2, lower panel), which together will benefit virus yield. Furthermore, the model predicts that a decrease in the vRNA synthesis rate, in the cRNA synthesis rate, and a delayed binding of NP to naked viral RNA, needed to form replication-competent vRNPs and cRNPs, will cause an increase in virus yield (Fig 1). These three predictions seem counterintuitive since they cause a slowdown of viral replication. On the other hand, however, this strongly suggests that there is an imbalance between viral RNA replication and viral protein synthesis. While the synthesis of viral genomes is saturated, i.e., the RNA synthesis rates are too high, the supply of viral proteins either needed to form RNPs (NP and polymerases) or needed for virus budding (HA and NA) represents a limiting step in A549 cells. Interestingly, Ueda and colleagues [23] made similar observations when comparing IAV growth in MDCK and A549 cells. While steps of viral replication were similar in both cell lines, A549 cells released fewer virions because both the maturation of glycoproteins and their transport to the plasma membrane were slower compared to MDCK cells. In line with that, parameter perturbation studies with the single cell model for MDCK cells [12] did not point to bottlenecks in viral transcription and translation (S4 Fig). Indeed, the MDCK-based model is more sensitive to a change in the vRNA synthesis rate compared to a change in the protein synthesis rate, while the A549-based model is highly sensitive to changes in the protein synthesis rate (S5 Fig).

### Analysis of influenza A virus replication in cell lines overexpressing a single host cell gene

We generated cell lines overexpressing host cell genes beneficial for virus replication previously determined by RNAi screening [9,13–15] and studies on virus-host cell interactions performed by other research groups [16–19].

Overall, the maximum virus yield was similar in all A549 cell populations. However, the engineered cell populations released more virus particles at earlier time points compared to the parental cell line during infection studies performed at low MOI. To assure that target genes were stably overexpressed, we confirmed the expression of the functionally linked reporter gene coding for eGFP by flow cytometric measurements during cell culture maintenance (S6 Fig). Furthermore, we determined relative expression levels of the transgenes in SGOs by RT-qPCR (S3 Table). Although the overall number of virus progeny produced by engineered cells was not significantly higher compared to the parental cell line, we could not exclude that intracellular mechanisms of virus replication had changed due to the modulation of host cell gene expression. To elucidate this in greater detail we investigated virus replication dynamics on the intracellular level both experimentally and computationally. With the help of the single cell model, we quantified the changes in key kinetic parameters by fitting to the available experimental data. In contrast to our initial model predictions, both nuclear import rate and viral mRNA synthesis rate were reduced in some SGOs compared to their parental A549 cell line. For instance, the viral mRNA synthesis rate in infected cells overexpressing the nuclear export factor NXF1 was only 60% of the one in parental A549 cells, which alone would lead to a reduction in virus yield by 50%. Still, the NXF1 SGO was the only cell line with a higher cell-specific virus yield when infected at MOI 1 (Fig 5D). The model can only capture these experimental data by an increase in the virus release rate. Hence, the improved virus release rescues virus yields such that despite the adverse changes in viral RNA synthesis, the SGOs release equal or slightly higher amounts compared to the parental A549 cell line. It was reported that inhibition of NXF1 in A549 cells impairs nuclear export of viral mRNAs encoding for NP as well as the surface proteins hemagglutinin (HA) and neuraminidase (NA) [18]. Upon NXF1 overexpression viral mRNA export might be improved, which may lead to an earlier onset of translation, such that viral surface proteins are available earlier compared to the parental A549 cell line, which is less efficient in protein maturation and trafficking [23]. In the single cell model these steps are not explicitly modeled but lumped into a joint release mechanism that depends on the availability of viral proteins and genome copies in the cytoplasm (S1 File, Equation 27). In addition, the importance of the virus release mechanism was also shown by initial model predictions (Fig 1) that identified virus assembly and budding as kinetic bottleneck of virus production.

The overall tendency that an increase in the virus release rate can compensate adverse changes in RNA synthesis steps can also be observed for infected CEACAM6 SGO cells. In contrast to NXF1, CEACAM6 is not directly involved in steps of RNA synthesis but seems to interact with newly synthesized viral NA proteins during infection, which activates the Src/Akt survival pathway in A549 cells as shown by Gaur and colleagues [16]. In the same study, CEACAM6-silenced A549 cells showed reduced levels of viral genome copies and proteins. However, in our study, the overexpression of CEACAM6 was not beneficial for IAV replication. Accordingly, temporal upregulation of CEACAM6 instead of high abundance seems to be crucial for cellular survival signaling during infection. Furthermore, members of the CEACAM family are already upregulated upon infection by different influenza virus strains, as recently also shown for CEACAM1 and CEACAM5 [24]. In particular, CEACAM1 induction triggers the innate antiviral host cell response by suppression of the translational machinery and limits viral spread [25]. Taken together, the ambivalent role of the CEACAM family and, in particular, the functional role of CEACAM6 in cellular survival pathways, may support the finding that the overexpression of CEACAM6 can be disadvantageous for IAV replication. Still, it is remarkable that CEACAM6 SGO cells release equal amounts of progeny virions compared to parental A549 cells, indicating that despite a certain inhibition of replication, the virus maintains a basal level of reproduction.

Except for SGOs NXF1 and CEACAM6, for which the nuclear import rate was slightly reduced (p ≤ 0.1, calculated by one-sided Gauss test), the nuclear import rate of vRNPs was similar in the other SGOs compared to parental A549 cells. For the PLD2 SGO, this was unexpected, since it is known that inhibition of PLD2 results in delayed virus entry and reduced viral titers [19]. Still, overexpressing PLD2 did neither improve virus entry nor virus release in our study. The only change in kinetic parameters, that was in agreement with initial model predictions (Fig 1) and should benefit virus yield, was the reduction of the cRNA synthesis rate to 50% compared to parental A549 cells. However, this alone would result in an increase of virus yield by only about 1.3-fold in simulations, a small improvement that is eliminated by a simultaneous decrease in the mRNA synthesis rate in PLD2 SGOs as determined from the experimental data.

The candidate FANCG interacts with the three viral polymerase subunits (PB2, PB1 and PA) and has a direct influence on polymerase activity according to a minigenome replicon assay using a vRNA-like reporter gene [17]. In this particular assay, it was demonstrated that a FANCG knockdown resulted in a decrease of polymerase activity by 50% while overexpression of FANCG showed a three-fold increase in polymerase activity. According to our initial model predictions, FANCG would have been the most promising candidate to improve virus yield, in particular, if the mRNA synthesis rate was increased (Fig 1). Surprisingly, all viral RNA species showed reduced levels in infected FANCG SGO cells. Although we have only performed two independent experiments to measure intracellular viral RNA levels in infected FANCG SGO cells, RNA copy numbers were lower compared to those in infected A549 cells in the same experiments as well as compared to the averaged RNA levels in A549 cells from all four independent experiments. Taken together, it seems that an overall increase of the viral polymerase activity results in imbalanced virus replication. Therefore, additional simulations were performed to test the effect of increasing all three or different combinations of the RNA synthesis rates simultaneously. However, by only increasing the vRNA synthesis rate, a reduction in virus yield is predicted (S7 Fig), while any other scenario leads to an increase in final yield in simulations (for instance see S8 and S9 Figs). Hence, our experimental observations together with the model-based analysis of this candidate are not in agreement with the study of Tafforeau and colleagues [17]. On the one hand, this may indicate that observations in an (artificial) minigenome replicon assay can only give hints towards changes in mechanisms and that the observation in the context of an infection, i.e., including additional regulatory steps of replication and availability of cellular and viral precursor molecules, can be contradictory. On the other hand, FANCG also has a beneficial function for the host cell, since it is involved in DNA repair mechanisms. We could, therefore, speculate that damage of cellular DNA induced by IAV infection [26] is reduced by overexpressing FANCG. However, we cannot exclude that FANCG plays a pro-viral role by interacting with the viral polymerase.

Similar to FANCG, also XAB2 is involved in DNA repair mechanisms, in particular, in transcription-coupled DNA repair [27]. XAB2 is a host restriction factor for IAV as well as for other viruses, e.g. West Nile virus, Vaccinia virus and HIV-1 [28]. In our study, however, the overexpression of this factor neither improved nor impaired viral reproduction.

### Analysis of virus release from cell lines overexpressing multiple host cell genes

In a few infected SGOs the change in various kinetic parameters should be beneficial for virus replication according to model predictions (Fig 1), e.g. a decrease in cRNA synthesis rate upon overexpression of FANCG, PLD2 or XAB2, or an increase in the virus release rate upon overexpression of CEACAM6, FANCG or NXF1. Using a Monte Carlo approach, we analyzed single cell simulations using randomized SGOs parameter sets to predict virus release of MGOs. This analysis revealed that the productivity of single cells follows a log-normal distribution with highly productive cells as rare events. This finding is supported by previous single-cell analyses performed by our group, which investigated the cell-specific productivity of MDCK cells infected by IAV. In particular, they demonstrated that there is a large variability in the productivity of individual cells and that only very few cells are highly productive (with up to 10-fold higher titers compared to the cell population average) [29,30]. Furthermore, the most recent study showed that single cell virus yields are log-normally distributed [30].

While MGO simulations suggest that particular combinations of genes have the potential to yield IAV titers similar to an *in silico* optimized cell line with an optimal virus release rate or M1 binding rate (open circles, Fig 7), we could not generate MGOs with an elevated overall HA titer. However, it has to be taken into account that all experimental data were acquired from cell populations of genetically modified cells with different combinations and expression levels of host cell genes. Thus, beneficial host cell factor combinations in individual cell clones might be masked. More extensive screening would be required to identify and isolate individual cell clones, which reflect the features predicted *in silico*.

### Applicability and limitations of the single cell model

The present version of the mathematical model of IAV replication is most suited to describe the impact of host cell factors that act directly on individual steps of the virus life cycle, e.g. factors that modulate the activity of the polymerases. The assumption that the influence of such factors also directly impacts kinetic parameters of the model enabled the identification of bottlenecks in virus replication that could be modulated by cell line engineering. Similar model-based approaches were performed previously by others to compare the replicative properties of different influenza virus strains [31,32] and virus replication with and without antiviral treatment [22,33]. While Binder and colleagues [34] compared low and high permissive host cells for hepatitis C virus replication that showed different intracellular basal concentrations of the same host cell factor, we applied the single cell model of IAV replication to quantify changes in key kinetic parameters of virus replication in cell lines overexpressing different host cell factors, which has not been reported before. Still, all these approaches have in common that they are solely computational, focusing on viral dynamics described by a fixed set of equations. As a result, in our study, similar ‘patterns’ of parameter changes were found for cell lines overexpressing host cell factors with very diverse functions, e.g. *k*^*Imp*^ ↓, $k_{V}^{Syn}\to$, $k_{C}^{Syn}\to$, $k_{M}^{Syn}↓$, $k_{M1}^{Bind}\to$ and *k*^*Rel*^ ↑ for both NXF1 and CEACAM6. Therefore, this model-based analysis can only provide indications regarding the general impact of an overexpressed host cell factor. Clearly, further in-depth characterization of the impact of host cell factors on individual steps of virus replication is required on the molecular level to fully comprehend the biological implications of parameter changes determined in the present work. To neglect details of cellular processes and pathways, e.g. cellular transcription and translation or immune response, may limit model predictions. On the contrary, the implementation of proposed functions of candidate host cell factors into the model may lead to biased interpretation of experimental data (self-fulfilling prophecies). More elaborate dynamic models on virus-host cell interactions should not only account for the viral life cycle but also include a mathematical description of the cellular pathways in which the considered host cell factors are involved. Yet, the biological knowledge about how most host cell factors impact the viral life cycle is too sparse and even controversial to be readily implemented into a mathematical framework. To elucidate this in more detail can only be accomplished through experiments which analyze changes in the viral life cycle together with the dynamics of host cell factors and the activity of the corresponding cellular pathways. Regarding the further improvement of quantitative models for intracellular virus replication, this will probably be one of the most challenging tasks to be performed over the next decades. Moreover, we model viral dynamics in an average infected cell and do not account for stochastic effects that play a role at low molecule numbers, i.e., for low MOI infections. We can therefore only estimate parameters from experimental infections performed at high MOI (MOI ≥ 1), which ensures that the majority of cells is infected simultaneously. Thus, the infection propagates synchronously in the cell population and virus release reaches steady state within 24 h. In these high MOI scenarios, replication can also be affected adversely by introducing a high number of non-infectious virions, e.g. defective interfering particles (DIPs). There is already a single cell model available that also describes the impact of DIPs on virus replication [35]. However, since the intracellular mechanisms of DIP interference remain elusive, we think that, the modeling of DIP propagation in engineered cell lines seems unreasonable but should be taken into account in future studies.

### Limitations of targets identified by RNAi screens and target validation studies

Usually, the significance of cellular targets identified from loss of function studies is limited, e.g. due to inefficient knockdown or off-target effects that lead to identification of false positives and false negatives (discussed in [36–38]). In our study, we therefore chose host cell factors relevant for IAV replication that were not only identified in RNAi screens, but have also been described previously in separate studies, except for XAB2. Still, the importance of these factors is mostly inferred from loss of function studies and we simply assume that if the knockdown of a host cell factor results in reduced virus growth, the overexpression of the same factor should improve virus replication. Overall, however, we found that most differences in both intracellular replication and progeny virus release were noticeable, but not statistically significant compared to parental A549 cells. Only when infected at MOI 10^−4^, engineered cell lines showed higher HA titers at early time points, while the HA titers of all cell lines were similar at time of harvest (72 h p.i.). Hence, we confirmed findings of screens for which changes in virus growth were evaluated at early time points (12–48 h p.i.) after infection at MOIs below one [9,13–15], where a single readout is useful to identify host cell factors that have a strong impact on viral dynamics. Such factors are very interesting in the context of antiviral treatment, for which the interference with virus replication early during infection might promote viral clearance in an *in vivo* system. Although they are required to complete the replication cycle successfully, such factors might not even limit viral replication at their basal expression level. Hence, their overexpression would not result in any measurable changes of intracellular mechanisms. To improve vaccine production, however, the expression of host cell factors should be increased which improve the maximum cell-specific productivity. For this purpose, screening designs should be re-considered to capture not only dynamics of virus growth but also virus yield at time of harvest. Since large scale high-throughput screens are costly, a first step might be the re-evaluation of already existing screens that considered multiple time points post infection (e.g. [10,39]). Recently, re-evaluation of primary data from various RNAi screens and different virus-host cell interaction studies, i.e., protein-protein interactions, transcriptomic and proteomic data, revealed and validated the impact of host cell factors on virus replication, that were previously unknown [40,41]. This highlights the importance of study design and subsequent bioinformatical analysis, which both strongly contribute to the identification of key host cell factors for intracellular virus replication and release.

Beyond that challenge, we have no indication regarding the optimal level of gene (over)-expression required to achieve a positive impact on virus growth, while avoiding off-target effects. In our study, we used lentiviral transduction without control of the integration site and assumed that cells, for which insertion of the overexpression constructs was beneficial, will propagate well in culture. Indeed, we saw that transduction of different host cell factors resulted in different levels of overexpression (S3 and S4 Tables) and surprisingly, that the cell line with a very low overexpression level of the host cell factor NXF1 was most promising with respect to early virus dynamics. In contrast, a high level of overexpression might stress the biosynthetic capacity of the cell, and result in a competition between expression of candidate genes and viral proteins. It is particularly known that the translation of viral proteins is the energetically most costly step of virus replication [42]. If the synthesis capacity of the cell is exploited by both overexpression of candidate genes and expression of viral proteins, cellular resources needed for virus growth might become limiting. Together, this might explain the observation that SGOs, in particular those showing high expression levels of the candidate gene, produce the same or only slightly higher virus yields compared to the parental A549 cell line. However, experimental proof would be needed to support these speculations. To better control overexpression levels, it might be worthwhile to explore other gene editing methods, e.g. recombinase-mediated cassette exchange [43] or CRISPR/Cas9 [44], for target validation studies. As discussed before, some host cell factors are already enriched upon infection and it might be also interesting to follow their expression levels over time and—based on that—design an inducible overexpression system to control supply of host cell factors in a temporal manner if this is needed for their function [45,46].

Finally, and as shown in a first attempt in this work, mechanistic models of the virus replication cycle are indispensable for evaluation and interpretation of infection data from engineered cell lines. Thus, we envision that screening approaches focusing on virus yield at harvest time points relevant in vaccine production supported by simulation studies using mathematical models for virus replication will enable the design of novel producer cell lines with the final goal to improve cell culture-based vaccine manufacturing. In addition, the combination of both, experimental and computational, approaches using data from well-defined experimental conditions will significantly deepen our understanding of intracellular mechanisms of virus-host cell interactions and support analyses of infectious diseases and virus transmission.

## Methods

### Model of intracellular influenza A virus replication in A549 cells

The model used in this study is a detailed mathematical description of intracellular IAV replication as published previously for adherent MDCK cells [12]. It accounts for key steps of the virus life cycle, using a set of ODEs to simulate virus entry, viral RNA and protein synthesis as well as virus assembly and progeny virus release. To predict virus replication and release for A549 cells, we assumed that these do not change mechanistically, but only show differences in their dynamics due to the change in the host cell system. To capture this, we performed a re-parameterization of nuclear vRNP import, viral replication, viral transcription and virus release based on experimental data obtained for infected A549 cells (S1 Fig). As an extension of the original version of this model, we also computed the percentage of nuclear vRNPs $frac_{Rnp}^{nuc}$ to fit measurements of nuclear vRNP import obtained by imaging flow cytometry (Fig 3).

$$Rnp^{cyt}=8V^{En}+Vp^{cyt}+Vp_{M1}^{cyt}$$ (1)

$$Rnp^{nuc}=Vp^{nuc}+Vp_{M1}^{nuc}$$ (2)

$$frac_{Rnp}^{nuc}=\left(\frac{Rnp^{nuc}}{Rnp^{nuc}+Rnp^{cyt}}\right)\cdot 100$$ (3)

The description of the complete mathematical model can be found in S1 File.

### Parameter estimation

Model parameters were estimated in two subsequent steps. First, the nuclear import rate *k*^*Imp*^ was estimated by fitting the simulated fraction of nuclear vRNPs $frac_{Rnp}^{nuc}$ to the mean of the relative fluorescence intensity (FI) of the nucleus $frac_{Int}^{nuc}$ determined by imaging flow cytometry (see Imaging flow cytometry and image analysis). For this, we assumed that the relative increase in FI of the nucleus is correlated directly to the increase in the fraction of nuclear vRNPs caused by nuclear import of the viral genomes which can be stained by a specific antibody (see Imaging flow cytometry and image analysis). In our experiments, we observed an offset for $frac_{Int}^{nuc}$ of approximately 50% at the time point of infection, which is related to the background signal of the nucleus and normally comprises between 40–60% of the cell’s area evaluated during image analysis. To account for this background signal, we applied an offset to the simulation values of $frac_{Rnp}^{nuc}$. Since offset values differed slightly between cell lines and showed occasionally high standard errors (Fig 3), we also estimated this offset value and optimized it with respect to the arithmetic mean and standard error of the first measurement point at zero h p.i. for each cell line. For fitting with parameter set *p*, we minimized the least-squares prediction error for all available data points at time point *t* weighted with the maximum measurement value (Eq 4).

$$\underset{p}{\min}\sum_{t_{0}}^{t_{end}}{\left(\frac{frac_{Rnp}^{nuc}(t)-frac_{Int}^{nuc}(t)}{\max(frac_{Int}^{nuc})}\right)}^{2}$$ (4)

After optimization of the nuclear import rate *k*^*Imp*^, we fitted our model to intracellular measurements of vRNA, cRNA and mRNA levels obtained from experiments at MOI 50 as well as to progeny particle numbers per cell for experiments at MOI 1. The corresponding set of kinetic parameters *p* was estimated simultaneously by minimizing the least-squares prediction error based on the decadic logarithm of all state variables *n*, whereby the error of each variable *i* was weighted with its maximum measurement value (Eq 5).

$$\underset{p}{\min}{\sum_{i}^{n}\sum_{t_{1}}^{t_{end}}\left(\frac{\log_{10}(prediction_{i}(t))-\log_{10}(data_{i}(t))}{\max(\log_{10}(data_{i}))}\right)}^{2}$$ (5)

To synchronize infection and facilitate parameter inference, we performed infections experiments at high MOI. Thus, due to the high virus concentration at time of infection, RT-qPCR already detected vRNA copies as soon as 1 h p.i. (Fig 4, panel 3). This value cannot be caused by an immediate uptake of all virions but rather stems from vRNAs inside virus particles and/or free vRNAs attached to the cells. Therefore, we applied the intracellular vRNA measurement value at 1 h p.i. as an offset to the simulated amount of vRNAs, as done before similarly in another modeling study of our group [22]. In contrast to this previous study, we did not apply offsets to viral mRNA and cRNA levels, as these RNA species are not part of virus particles and are usually not present in the seed virus supernatant. In particular, cRNA levels at 1 h p.i. were below or close to one copy per cell and have no significant impact on simulation results. Finally, approximately 10 copies of mRNA per cell were detected at 1 h p.i. Since mRNA synthesis starts as early as vRNPs reach the nucleus, these mRNAs are a product of primary transcription and cannot be considered as a plain mRNA offset.

The parameter distributions were determined by parametric bootstrapping performing multiple model fits to 3000 random resamples from the experimental data according to their mean and standard deviation, as detailed elsewhere [47]. We set the medians of the resulting parameter distributions as parameter optima to perform simulations. For the SGO candidate FANCG, only duplicate measurements of the intracellular viral RNA were available. Therefore, we considered a relative standard error of 50%, which was the average relative standard error of all other RNA measurements performed in this study.

### *In silico* analysis of cell lines overexpressing a single gene

The modeling approaches in this work are based on the simplifying assumption that each step of the virus life cycle is directly dependent on the presence of relevant host cell factors and that their influence is changed by manipulating the expression of the corresponding genes. For instance, if a host cell factor crucial for viral RNA synthesis is knocked down, the efficiency of vRNA synthesis is reduced as well, resulting in a lower vRNA synthesis rate. When the same host cell factor is overexpressed, RNA replication is enhanced, which results in a higher vRNA synthesis rate. Using this assumption, we determined the optimal value for individual kinetic parameters of the model by maximizing the number of released progeny virions at 24 h p.i. To predict biologically reasonable values, we constrained the parameter search by a lower bound of factor 0.2 and an upper bound of factor 5 of the original parameter values, respectively.

### *In silico* analysis of cell lines overexpressing multiple host cell genes

In this study, lentiviruses were used to modify the expression of host cell factors relevant for IAV replication. Gene editing constructs delivered by lentiviruses are integrated randomly at different chromosomal locations with different transcriptional activity (reviewed in [48]). Therefore, we can anticipate that individual cells within a transduced cell population will show heterogeneity with respect to levels of relative overexpression.

Consequently, the transduction of more than one overexpression construct leads to an even larger heterogeneity in gene expression levels. To simulate IAV production of MGOs, we account for the non-targeted integration of multiple gene constructs by randomly compiling new parametrizations of the single cell model. More precisely, we assume that IAV can propagate in an individual cell of an MGO population with random combinations of kinetic parameters as determined before in detailed characterizations of SGO populations. In addition, to account for the adverse impacts by off-target effects, we also included the parameter set of the unmodified parental A549 cell line for randomization.

To facilitate the interpretation of simulation results for MGOs, we simulated IAV replication with randomly assembled parameter sets for a single cell infection at MOI 1 for 48 h p.i. In a next step, we evaluated each simulation with respect to maximum virus yield and the time point of first virus release, i.e., the time p.i. when the first simulated virus particle was released (*V*^*Rel*^≥1).

To assure that a sufficient number of simulations was performed that would allow reasonable conclusions on MGO single cell infections, we repeated simulations with randomized parameter sets n times until the relative deviation between the mean of n-1 and mean of n simulated maximum virus yields reached 1 x 10^−8^.

### Simulation and computation

Model equations were solved numerically using the CVODE routine from SUNDIALS [49] on a Linux-based system. All model parameter values and initial conditions are given in S5 and S6 Tables. Model files and experimental data were handled within the Systems Biology Toolbox 2 [50] for MATLAB (version 8.0.0.783 R2012b). Parameter values were estimated by the least-squares method as explained before (see Parameter estimation), using the global stochastic optimization algorithm fSSm [51].

### Statistics

To determine the significance level of differences in parameter distributions between parental A549 and engineered cell lines (SGOs) we performed a one-sided Z test (Gauss test) with mean $\bar{p}$ and variance *σ*^2^ taken from the empiric parameter distributions to compute the following test statistic Z:

$$Z=\frac{\bar{p_{A549}}-\bar{p_{SGO}}}{\sqrt{\frac{\sigma_{A549}^{2}}{n}+\frac{\sigma_{SGO}^{2}}{m}}}$$ (6)

For this, the variance is usually normalized by the sample sizes n and m. However, we set the sample sizes to 1 instead of 3000 for the number of bootstrapped resamples, since the artificially high sample size is otherwise biasing the test result. This was also done previously by others to compare parameters of mutant to wild type viruses [31]. Following their approach, we generally assume that parameters are normally distributed. Only if parameter distributions followed a log-normal form, namely the vRNA synthesis rate $k_{V}^{Syn}$ and the virus release rate *k*^*Rel*^, the test statistics were calculated based on the decadic logarithm of these parameters.

To determine statistical significance in differences of measurements from SGOs and the parental A549 cell line, the Kruskal-Wallis test was performed as available in MATLAB (version 8.0.0.783 R2012b).

### Lentiviral vectors and transduction of A549 cells

Human cDNAs encoding CEACAM6, XAB2, FANCG, NXF1 and PLD2 were purchased from the I.M.A.G.E consortium. The cDNA sequences were amplified by PCR and cloned into the bicistronic lentiviral vector pLV-X-GFPneo. This vector was derived from pLVtTRKRAB-Red [52] by integrating the fusion gene of GFP and neomycin phosphotransferase in the second cistron. Lentiviral vectors were produced by transfecting HEK293T cells with the pLV-X-GFPneo and the lentiviral helper plasmids coding for gag-pol, Rev and VSV-G using the calcium phosphate transfection protocol as detailed in [53]. The supernatant was collected two days post transfection, filtered (0.45 μm), titrated and stored at -80°C.

At the day of transduction, the virus supernatant was supplemented with polybrene (8 μg/mL) and added to 1 x 10^5^ A549 cells. After 6 h the virus was removed and cells were cultured in Dulbecco’s Modified Eagle Medium (DMEM, GIBCO) with 10% (v/v) fetal calf serum (FCS, Sigma-Aldrich). On the day after infection, selection with neomycin was started (1 mg/mL G418). G418-resistant cell populations were maintained as transduced populations. FACS was performed to enrich cell populations expressing eGFP.

For generation of MGOs, cells were transduced with two cocktails of two to three different lentivirus stocks each on two consecutive days using MOI 1 per virus.

### Cell culture and virus infection

Parental A549 cells [54,55] and transduced A549 cell lines were maintained in DMEM with non-essential amino acids, 10% (v/v) FCS at 37°C and 5% CO_2_ atmosphere. Prior to infection, cells were washed twice with phosphate buffered saline (PBS), detached and counted using a Vi-CELL XR^TM^ (Beckman Coulter). Subsequently, 0.4 x 10^6^ cells per well were seeded into multiple 12-well plates and incubated overnight. Infection was performed with an A549-adpated seed virus preparation of influenza virus A/Puerto Rico/8/34 (#3138, Robert Koch Institute Berlin) which had an infectious virus titer of 1.08 x 10^8^ virions per mL as determined by TCID_50_ (see [56] for detailed description of the TCID_50_ assay). For infection, cells were washed twice with PBS and virus was added together with serum-free cell culture medium containing trypsin (#T7409, Sigma-Aldrich) at a concentration of 1 x 10^-4^ units per cell. To support synchronous infection of cells, experiments were carried out at MOI 50 in a reduced volume of 300 μL per well. After 30 min, 700 μL DMEM was added to compensate for liquid losses through evaporation. To investigate the nuclear import of viral genomes, cells were treated with the translation inhibitor CHX (Sigma Aldrich). For this, cells were incubated for 1 h in serum-free culture medium at a CHX concentration of 100 μg per mL. Then, infection was performed by replacing the supernatant with serum-free culture medium containing seed virus, trypsin and CHX.

### Virus quantification

The amount of total virus particles in the supernatant of infected cells was determined by the hemagglutination assay as described by Kalbfuss and colleagues [57]. The virus titer measured as log_10_ HA units per test volume (log_10_ HAU per 100 μL) can be used to estimate the concentration of hemagglutinating particles *c*_*virus*_ with

$$c_{virus}=c_{Ery}\cdot 10^{\left(\log_{10}HAU/100\mu L\right)},$$ (7)

assuming that one virus particle per erythrocyte is sufficient to cause agglutination [58,59], where *c*_*Ery*_ denotes the concentration of chicken erythrocytes added for hemagglutination (2 x 10^7^ cells per mL). The number of virions released per cell was assessed by dividing the virus concentration by the maximum viable cell count obtained in each experiment.

### Real-time RT-qPCRs

Viral and cellular RNA were purified from cells using the extraction kit ‘NucleoSpin RNA’ (Macherey-Nagel) according to the manufacturer’s instructions. To quantify intracellular viral RNA levels of segment 5 (encoding viral nucleoprotein, NP) polarity- and gene-specific tagged primers (listed in S7 Table) were used for reverse transcription to distinguish between the three different RNA species of the IAV genome (as detailed in [60]). Reference standards were synthesized *in vitro* using a specific set of primers (listed in S8 Table) and supplemented with 350 ng of RNA from A549 cells to mimic intracellular conditions. In order to determine relative overexpression levels of host cell genes, mRNA of uninfected A549 cells was reverse transcribed using Oligo(dT) primers (listed in S9 Table).

For both, viral and cellular RNA, real time RT-pPCR was performed using the Rotor-Gene SYBR Green PCR Kit and Rotorgene Q (Qiagen) according to the manufacturer’s instructions. The calculation on viral RNA molecule numbers per cell was performed as described in [60]. Relative expression levels of host cell genes in SGOs and MGOs compared to the parental A549 cells were calculated by the $2^{-\Delta \Delta C_{T}}$ method, using 18S rRNA as a calibrator [61].

### Imaging flow cytometry and image analysis

For the analysis of nuclear vRNP import, 1 x 10^6^ infected A549 cells were fixated with paraformaldehyde (PFA) at a final concentration of 1% (w/v) for 30 min on ice. Subsequently, samples were transferred to reaction tubes, cells pelleted by centrifugation (8 min, 300 x g, 4°C) and resuspended in 70% ice-cold ethanol before storage at -20°C.

For vRNP and DAPI staining, stored samples were centrifuged (8 min, 300 x g, 4°C) and the cell pellet was resuspended in wash buffer (PBS, 2% (w/v) glycine, 0.1% (w/v) bovine serum albumin (BSA)) and centrifuged as before. Afterwards, the cell pellets were resuspended in 150 μL wash buffer, transferred to 96-well plates and centrifuged once more. Next, cell pellets were resuspended in 25 μL blocking buffer (wash buffer with 1.1% (w/v) BSA) and incubated for 30 min at 37°C. After a final washing step with 200 μL wash buffer, cells were resuspended in 25 μL antibody solution and incubated for 1 h at 37°C. The anti-NP antibody mAb61A5 that preferentially binds oligomerized NP as present in the vRNP complex, was kindly provided by Fumitaka Momose [62]. Upon incubation, cells were washed three times with wash buffer and afterwards 25 μL of Alexa Fluor 647-conjugated polyclonal goat anti-mouse antibody (Life Technologies, #A21235) solution was added to the cells and incubated for 1 h at 37°C. Both the primary and secondary antibody were used at a dilution of 1:500 in wash buffer. Finally, cells were washed three times and the cell pellet was resuspended in 30 μL wash buffer with 2% (v/v) DAPI (Roth, 143 μM stock solution) for nuclear staining. After 5 min of incubation in the dark at room temperature, cells were measured using the ImageStream X Mark II (Amnis, EMD, Millipore) together with the INSPIRE software. For each sample 10,000 single cells were analyzed using the 60x magnification and the 375 nm and 642 nm lasers for excitation of DAPI and vRNP antibody, respectively. Channels 1 (DAPI signal, CH1) and 5 (Alexa Flour 647, CH5) were acquired together with channel 6 (CH6), which records the bright field (BF) image. The laser powers were adjusted according to the value of the ‘raw max pixel’ feature that should be in the range between 200 and 1500 for single-stained positive controls. Furthermore, 1000 single positive cells were measured to adjust the compensation settings.

To evaluate the localization of vRNPs only double positive single cells in focus were selected for analysis. In order to distinguish between nucleus and the whole cell, a nucleus mask and a cell mask were defined according to the DAPI signal on CH1 and the BF image on CH6, respectively (examples are shown in S10 Fig). To determine the relative fluorescence intensity of the vRNP signal (CH5) located in the nucleus, the intensity of the vRNP signal within the nucleus mask was divided by the intensity of the vRNP signal within the whole cell mask.

## Supporting information

S1 Table Summary of *in silico* optimized kinetic parameters and corresponding model response according to the analysis shown in Fig 1. (DOCX)

S2 Table Uniprot identifier, names and functions of host cell genes used in this study. (DOCX)

S3 TableOverexpression level of host cell genes in cell lines overexpressing one of the indicated genes as determined by $2^{-\Delta \Delta C_{T}}$ method.(DOCX)

S4 TableOverexpression level of host cell genes in cell lines overexpressing multiple genes (MGOs) as determined by $2^{-\Delta \Delta C_{T}}$ method.(DOCX)

S5 Table Comparison of key kinetic parameters of influenza A virus replication in parental A549 cells and A549 cells overexpressing selected host cell factors (SGOs). (DOCX)

S6 Table Parameters used for the simulation of intracellular IAV replication. (DOCX)

S7 Table Primer sets for reverse transcription and real-time RT-qPCR for segment 5 of A/PR/8/34 (H1N1). (DOCX)

S8 Table Primer sets for the generation of RNA reference standards for A/PR/8/34 (H1N1) segment 5. (DOCX)

S9 Table Primer sets for PCR of host cell mRNA. (DOCX)

S1 Fig Comparison of simulations of intracellular influenza A virus replication in MDCK and parental A549 cells. Model fit (blue lines) to experimental data (blue symbols) for A549 and simulations for MDCK cells (brown lines) are shown, respectively. (A, B) Intracellular dynamics of viral RNA for a simulated infection at MOI 50 for vRNA and cRNA (circles, solid line) as well as for mRNA (squares, dashed line) in A549 cells and MDCK cells. (C) Nuclear import of viral genomes in CHX-treated cells for a simulated infection at MOI 50. For better comparison, the simulated fraction of nuclear vRNPs in MDCK cells was compressed with respect to the vRNP offset of A549 cells. (D) Cell-specific virus release for a simulated infection at MOI 1.
(TIF)

S2 Fig Comparison of parameter distributions for different A549 cell lines. Decadic logarithm of parameter values for fitting 3000 resamplings of the available experimental data obtained from SGOs. Shown are median (red solid line), first and third quartile (blue box), maximum values (whiskers) and outliers (red crosses). Blue dashed lines represent the median of the respective parameter in parental A549 cells. Experimental data for estimating the nuclear vRNP import rate in cycloheximide-treated cells (A) were resampled separately from those used for simultaneous estimation of vRNA (B), cRNA (C), mRNA (D), M1 binding (E) and virus release rate (F) in conventional infection experiments (without CHX treatment).
(TIF)

S3 Fig Simulated virus release dynamics of MGO CFNPX and A549 cells. Light blue area shows the mean and standard deviation of released virions from approximately 2 x 10^4^ simulations with randomized parameter sets, for a simulated infection at MOI 1. Infection of parental A549 cells, the transduction control and SGOs were simulated with the optimized parameter sets as determined in the present study (colors according to legend).
(TIF)

S4 Fig Virus release dynamics in response to *in silico* manipulation of gene expression of host cell factors in MDCK cells. We assume that efficiency of individual steps in the virus life cycle is directly dependent on host cell factors and that their influence is changed upon knockdown or overexpression of the corresponding gene. We simulated manipulation of gene expression by perturbing the corresponding kinetic parameters in the IAV replication model for MDCK cells established previously by our group [12] according to the approach presented for A549 cells (Fig 1). For the most important steps, virus release of parental MDCK cells (blue solid line) and the engineered cell line (brown solid line) are shown for a simulated infection at MOI 1. Colors indicate whether perturbation of the indicated step improved final virus yield at 24 h p.i. by at least two-fold (green), or had no impact (red). Scheme of IAV replication adapted from [22].
(TIF)

S5 Fig Fold change in final virus yield in response to parameter perturbations. We simulated manipulation of vRNA synthesis (column 1), viral protein synthesis (column 2) and the binding of the matrix protein 1 (M1) to nuclear vRNPs (column 3) by perturbing the corresponding kinetic parameters in the IAV replication model for both A549 cells established in the present study (upper panel) and for MDCK cells established previously by our group [12] (lower panel). Shown are the fold changes of the virus yield at 24 h p.i. in response to the fold changes in the corresponding parameters (black solid lines) with respect to the simulation of the parental cell lines. For every parameter analysis the simulation read out for the parental cell line (black open circle) and the optimal cell line (red cross) is marked.
(TIF)

S6 Fig Flow cytometry measurement of eGFP from parental and transduced A549 cell lines during cell culture maintenance. PFA-fixated cells were measured by imaging flow cytometry using the 488 nm laser. The eGFP signal of single cells in focus was evaluated using the mean FI (mean pixel feature) of channel 2 (CH02) and visualized as histograms for parental A549 cells (A), the transduction control (B) and A549 cells overexpressing one of the following host cell factors: NXF1 (C), CEACAM6 (D), FANCG (E), PLD2 (F), XAB2 (G).
(TIF)

S7 Fig Simulation of viral components in parental A549 cells and an *in silico* A549 cell line with changed parameters according to findings for the impact of FANCG on viral polymerase activity, proposed by Tafforeau and colleagues [17]. Virus particle release (A) and dynamics of intracellular vRNA (B), cRNA (C) and mRNA (D) if overexpression of FANCG causes a three-fold increase in the vRNA synthesis rate.
(TIF)

S8 Fig Simulation of viral components in parental A549 cells and an *in silico* A549 cell line with changed parameters according to findings for the impact of FANCG on viral polymerase activity, proposed by Tafforeau and colleagues [17]. Virus particle release (A) and dynamics of intracellular vRNA (B), cRNA (C) and mRNA (D) if overexpression of FANCG causes a three-fold increase in the synthesis rates for viral vRNA, cRNA and mRNA.
(TIF)

S9 Fig Simulation of viral components in parental A549 cells and an *in silico* A549 cell line with changed parameters according to findings for the impact of FANCG on viral polymerase activity, proposed by Tafforeau and colleagues [17]. Virus particle release (A) and dynamics of intracellular vRNA (B), cRNA (C) and mRNA (D) if overexpression of FANCG causes a three-fold increase in the mRNA synthesis rates.
(TIF)

S10 Fig Definition of nucleus and whole cell mask for image analysis of vRNP localization in infected A549 cells. The nucleus mask was defined with the help of the “morphology” feature on CH1 (DAPI signal) and the whole cell mask with the “object” feature on CH6 (bright field).
(TIF)

S1 File List of the ODE model equations used in the present study to simulate IAV replication in a single cell. (DOCX)

S2 File Experimental data. Contains measurements on nuclear import of viral genomes, intracellular viral RNA and virus release.
(XLSX)

## References

[1] WHO. FAQs WHO Estimate of Respiratory Deaths due to Seasonal Influenza. 2018; Available: http://www.who.int/influenza/surveillance_monitoring/bod/en/

[2] Shaw ML, Stertz S. Role of Host Genes in Influenza Virus Replication Current Topics in Microbiology and Immunology. Berlin, Heidelberg: Springer; 2017 pp. 1–39. 10.1007/82 28643205

[3] Müller KH, Kakkola L, Nagaraj AS, Cheltsov A V, Anastasina M, Kainov DE. Emerging cellular targets for influenza antiviral agents. Trends Pharmacol Sci. 2012;33: 89–99. 10.1016/j.tips.2011.10.004 22196854

[4] Watanabe T, Kawakami E, Shoemaker JE, Lopes TJS, Matsuoka Y, Tomita Y, et al Influenza Virus-Host Interactome Screen as a Platform for Antiviral Drug Development. Cell Host Microbe. 2014;16: 795–805. 10.1016/j.chom.2014.11.002 25464832 PMC4451456

[5] Tripathi S, Batra J, Lal SK. Interplay between influenza A virus and host factors: targets for antiviral intervention. Archives of Virology. 2015 pp. 1877–1891. 10.1007/s00705-015-2452-9 26016443

[6] Yip TF, Selim ASM, Lian I, Lee SMY. Advancements in Host-Based Interventions for Influenza Treatment. Front Immunol. 2018;9 10.3389/fimmu.2018.01547 30042762 PMC6048202

[7] van der Sanden SMG, Wu W, Dybdahl-Sissoko N, Weldon WC, Brooks P, Donnell JO, et al Engineering Enhanced Vaccine Cell Lines To Eradicate Vaccine-Preventable Diseases: the Polio End Game. J Virol. 2016;90: 1694–1704. 10.1128/JVI.01464-15 26581994 PMC4733985

[8] Hoeksema F, Karpilow J, Luitjens A, Lagerwerf F, Havenga M, Groothuizen M, et al Enhancing viral vaccine production using engineered knockout vero cell lines–A second look. Vaccine. 2018;36: 2093–2103. 10.1016/j.vaccine.2018.03.010 29555218 PMC5890396

[9] Karlas A, Machuy N, Shin Y, Pleissner K-P, Artarini A, Heuer D, et al Genome-wide RNAi screen identifies human host factors crucial for influenza virus replication. Nature. 2010;463: 818–22. 10.1038/nature08760 20081832

[10] König R, Stertz S, Zhou Y, Inoue A, Hoffmann HH, Bhattacharyya S, et al Human host factors required for influenza virus replication. Nature. 2010;463: 813–817. 10.1038/nature08699 20027183 PMC2862546

[11] Watanabe T, Watanabe S, Kawaoka Y. Cellular networks involved in the influenza virus life cycle. Cell Host Microbe. 2010;7: 427–439. 10.1016/j.chom.2010.05.008 20542247 PMC3167038

[12] Heldt FS, Frensing T, Reichl U. Modeling the Intracellular Dynamics of Influenza Virus Replication To Understand the Control of Viral RNA Synthesis. Journal of Virology. 2012 pp. 7806–7817. 10.1128/JVI.00080-12 22593159 PMC3421648

[13] Hao L, Sakurai A, Watanabe T, Sorensen E, Nidom CA, Newton MA, et al Drosophila RNAi screen identifies host genes important for influenza virus replication. Nature. 2008;454: 890–893. 10.1038/nature07151 18615016 PMC2574945

[14] Shapira SD, Gat-Viks I, Shum BOV, Dricot A, de Grace MM, Wu L, et al A Physical and Regulatory Map of Host-Influenza Interactions Reveals Pathways in H1N1 Infection. Cell. 2009;139: 1255–1267. 10.1016/j.cell.2009.12.018 20064372 PMC2892837

[15] Brass AL, Huang I, Benita Y, John SP, Krishnan MN, Feeley EM, et al The IFITM Proteins Mediate Cellular Resistance to Influenza A H1N1 Virus, West Nile Virus, and Dengue Virus. Cell. 2009;139: 1243–1254. 10.1016/j.cell.2009.12.017 20064371 PMC2824905

[16] Gaur P, Ranjan P, Sharma S, Patel JR, Bowzard JB, Rahman SK, et al Influenza A virus neuraminidase protein enhances cell survival through interaction with carcinoembryonic antigen-related cell adhesion molecule 6 (CEACAM6) protein. J Biol Chem. 2012;287: 15109–15117. 10.1074/jbc.M111.328070 22396546 PMC3340274

[17] Tafforeau L, Chantier T, Pradezynski F, Pellet J, Mangeot PE, Vidalain P, et al Generation and comprehensive analysis of an influenza virus polymerase cellular interaction network. J Virol. 2011;85: 13010–8. 10.1128/JVI.02651-10 21994455 PMC3233135

[18] Larsen S, Bui S, Perez V, Mohammad A, Medina-Ramirez H, Newcomb LL. Influenza polymerase encoding mRNAs utilize atypical mRNA nuclear export. Virol J. 2014;11: 1–11. 10.1186/1743-422X-11-1 25168591 PMC4158059

[19] Oguin TH, Sharma S, Stuart AD, Duan S, Scott SA, Jones CK, et al Phospholipase D facilitates efficient entry of influenza virus, allowing escape from innate immune inhibition. J Biol Chem. 2014;289: 25405–25417. 10.1074/jbc.M114.558817 25065577 PMC4162146

[20] Stegmann T, Wey J, Bartoldus I, Schoen P, Bron R, Ortiz A, et al Evaluation of Viral Membrane Fusion Assays. Comparison of the Octadecylrhodamine Dequenching Assay with the Pyrene Excimer Assay. Biochemistry. 1993;32: 11330–11337. 10.1021/bi00093a009 8218197

[21] Kawakami E, Watanabe T, Fujii K, Goto H, Watanabe S, Noda T, et al Strand-specific real-time RT-PCR for distinguishing influenza vRNA, cRNA, and mRNA. J Virol Methods. 2011;173: 1–6. 10.1016/j.jviromet.2010.12.014 21185869 PMC3049850

[22] Heldt FS, Frensing T, Pflugmacher A, Gröpler R, Peschel B, Reichl U. Multiscale Modeling of Influenza A Virus Infection Supports the Development of Direct-Acting Antivirals. PLoS Comput Biol. 2013;9 10.1371/journal.pcbi.1003372 24278009 PMC3836700

[23] Ueda M, Yamate M, Du A, Daidoji T. Maturation efficiency of viral glycoproteins in the ER impacts the production of influenza A virus. Virus Res. 2008;136: 91–97. 10.1016/j.virusres.2008.04.028 18550190

[24] Sikora D, Rocheleau L, Brown EG, Pelchat M. Influenza A virus cap-snatches host RNAs based on their abundance early after infection. Virology. 2017;509: 167–177. 10.1016/j.virol.2017.06.020 28646652

[25] Vitenshtein A, Weisblum Y, Hauka S, Halenius A, Oiknine-Djian E, Tsukerman P, et al CEACAM1-Mediated Inhibition of Virus Production. Cell Rep. 2016;15: 2331–2339. 10.1016/j.celrep.2016.05.036 27264178 PMC4914772

[26] Li N, Parrish M, Chan TK, Yin L, Rai P, Yoshiyuki Y, et al Influenza infection induces host DNA damage and dynamic DNA damage responses during tissue regeneration. Cell Mol life Sci. 2015;72: 2973–88. 10.1007/s00018-015-1879-1 25809161 PMC4802977

[27] Kuraoka I, Ito S, Wada T, Hayashida M, Lee L, Saijo M, et al Isolation of XAB2 complex involved in pre-mRNA splicing, transcription, and transcription-coupled repair. J Biol Chem. 2008;283: 940–950. 10.1074/jbc.M706647200 17981804

[28] Thakur A, Qureshi A, Kumar M. VhfRNAi: A web-platform for analysis of host genes involved in viral infections discovered by genome wide RNAi screens. Mol Biosyst. Royal Society of Chemistry; 2017;13: 1377–1387. 10.1039/c6mb00841k 28561835

[29] Heldt FS, Kupke SY, Dorl S, Reichl U, Frensing T. Single-cell analysis and stochastic modelling unveil large cell-to-cell variability in influenza A virus infection. Nat Commun. 2015;6: 8938 10.1038/ncomms9938 26586423 PMC4673863

[30] Kupke SY, Riedel D, Frensing T, Zmora P, Reichl U. A novel type of influenza A virus-derived defective interfering particle with nucleotide substitutions in its genome. J Virol. 2018; JVI.01786–18. 10.1128/JVI.01786-18 30463972 PMC6364022

[31] Pinilla LT, Holder BP, Abed Y, Boivin G, Beauchemin CAA. The H275Y Neuraminidase Mutation of the Pandemic A/H1N1 Influenza Virus Lengthens the Eclipse Phase and Reduces Viral Output of Infected Cells, Potentially Compromising Fitness in Ferrets. J Virol. 2012;86: 10651–10660. 10.1128/JVI.07244-11 22837199 PMC3457267

[32] Simon PF, De La Vega MA, Paradis É, Mendoza E, Coombs KM, Kobasa D, et al Avian influenza viruses that cause highly virulent infections in humans exhibit distinct replicative properties in contrast to human H1N1 viruses. Sci Rep. Nature Publishing Group; 2016;6: 1–13. 10.1038/s41598-016-0001-8 27080193 PMC4832183

[33] Clausznitzer D, Harnisch J, Kaderali L. Multi-scale model for hepatitis C viral load kinetics under treatment with direct acting antivirals. Virus Res. 2015;218: 96–101. 10.1016/j.virusres.2015.09.011 26409026

[34] Binder M, Sulaimanov N, Clausznitzer D, Schulze M, Hüber CM, Lenz SM, et al Replication Vesicles are Load- and Choke-Points in the Hepatitis C Virus Lifecycle. PLoS Pathog. 2013;9 10.1371/journal.ppat.1003561 23990783 PMC3749965

[35] Laske T, Heldt FS, Hoffmann H, Frensing T, Reichl U. Modeling the intracellular replication of influenza A virus in the presence of defective interfering RNAs. Virus Res. 2016;213: 90–99. 10.1016/j.virusres.2015.11.016 26592173

[36] Hirsch AJ. The use of RNAi-based screens to identify host proteins involved in viral replication. Future Microbiol. 2010;5: 303–311. 10.2217/fmb.09.121 20143951 PMC2864646

[37] Stertz S, Shaw ML. Uncovering the global host cell requirements for influenza virus replication via RNAi screening. Microbes Infect. 2011;13: 516–525. 10.1016/j.micinf.2011.01.012 21276872 PMC3071880

[38] König R, Stertz S. Recent strategies and progress in identifying host factors involved in virus replication. Curr Opin Microbiol. 2015;26: 79–88. 10.1016/j.mib.2015.06.001 26112615 PMC7185747

[39] Zhou Z, Cao M, Guo Y, Zhao L, Wang J, Jia X, et al Fragile X mental retardation protein stimulates ribonucleoprotein assembly of influenza A virus. Nat Commun. 2014;5: 3259 10.1038/ncomms4259 24514761

[40] Chasman D, Walters KB, Lopes TJS, Eisfeld AJ, Kawaoka Y, Roy S. Integrating Transcriptomic and Proteomic Data Using Predictive Regulatory Network Models of Host Response to Pathogens. PLOS Comput Biol. 2016;12: e1005013 10.1371/journal.pcbi.1005013 27403523 PMC4942116

[41] Tripathi S, Pohl MO, Zhou Y, Rodriguez-Frandsen A, Wang G, Stein DA, et al Meta- and Orthogonal Integration of Influenza “OMICs” Data Defines a Role for UBR4 in Virus Budding. Cell Host Microbe. 2015;18: 723–735. 10.1016/j.chom.2015.11.002 26651948 PMC4829074

[42] Mahmoudabadi G, Milo R, Phillips R. Energetic cost of building a virus. Proc Natl Acad Sci U S A. 2017/05/16. National Academy of Sciences; 2017;114: E4324–E4333. 10.1073/pnas.1701670114 28512219 PMC5465929

[43] Wirth D, Gama-Norton L, Riemer P, Sandhu U, Schucht R, Hauser H. Road to precision: recombinase-based targeting technologies for genome engineering. Curr Opin Biotechnol. 2007;18: 411–419. 10.1016/j.copbio.2007.07.013 17904350

[44] Wang H, La Russa M, Qi LS. CRISPR/Cas9 in Genome Editing and Beyond. Annu Rev Biochem. 2016;85: 227–264. 10.1146/annurev-biochem-060815-014607 27145843 PMC13384723

[45] Gossen M, Bujard H. Tight control of gene expression in mammalian cells by tetracycline-responsive promoters. Proc Natl Acad Sci U S A. 1992;89: 5547–51. 10.1073/pnas.89.12.5547 1319065 PMC49329

[46] Weber W, Fussenegger M. Inducible gene expression in mammalian cells and mice In: Balbás P, Lorence A, editors. Methods in molecular biology. Second Edi. Totowa, New Jersey: HUMANA Press Inc; 2004 pp. 451–466. 10.1385/1-59259-774-2:451 15269442

[47] Efron B, Tibshirani R. Bootstrap Methods for Standard Errors, Confidence Intervals, and Other Measures of Statistical Accuracy. Stat Sci. 1986;1: 54–77.

[48] Bushman F, Lewinski M, Ciuffi A, Barr S, Leipzig J, Hannenhalli S, et al Genome-wide analysis of retroviral DNA integration. Nat Rev Microbiol. 2005;3: 848–858. 10.1038/nrmicro1263 16175173

[49] Cohen SD, Hindmarsh AC. CVODE, a stiff/nonstiff ODE solver in C. Comput Phys. 1996;10: 138–143. 10.1063/1.4822377

[50] Schmidt H, Jirstrand M. Systems Biology Toolbox for MATLAB: A computational platform for research in systems biology. Bioinformatics. 2006;22: 514–515. 10.1093/bioinformatics/bti799 16317076

[51] Egea JA, Rodríguez-Fernández M, Banga JR, Martí R. Scatter search for chemical and bio-process optimization. J Glob Optim. 2007;37: 481–503. 10.1007/s10898-006-9075-3

[52] Wiznerowicz M, Trono D. Conditional Suppression of Cellular Genes: Lentivirus Vector-Mediated Drug-Inducible RNA Interference. J Virol. 2003;77: 8957–8961. 10.1128/JVI.77.16.8957-8961.2003 12885912 PMC167245

[53] May T, Eccleston L, Herrmann S, Hauser H, Goncalves J, Wirth D. Bimodal and hysteretic expression in mamalian cells from a synthetic gene circuit. PLoS One. 2008;3: 1–7. 10.1371/journal.pone.0002372 18523635 PMC2394661

[54] Giard DJ, Aaronson SA, Todaro GJ, Arnstein P, Kersey JH, Dosik H, et al In vitro cultivation of human tumors: establishment of cell lines derived from a series of solid tumors. J Natl Cancer Inst. 1973;51: 1417–1423. 10.1093/jnci/51.5.1417 4357758

[55] Lieber M, Smith B, Szakal A, Nelson-Rees W, Torado G. A continuous tumor-cell line from a human lung carcinoma with properties of type II alveolar epithelial cells. Int J Cancer. 1976;17: 62–70. 175022 10.1002/ijc.2910170110

[56] Genzel Y, Reichl U. Vaccine production—state of the art and future needs in upstream processing In: Pörtner R, editor. Animal Cell Biotechnology: Methods and Protocols. Totowa, New Jersey: HUMANA Press Inc; 2007 pp. 457–473.

[57] Kalbfuss B, Knöchlein A, Kröber T, Reichl U. Monitoring influenza virus content in vaccine production: Precise assays for the quantitation of hemagglutination and neuraminidase activity. Biologicals. 2008;36: 145–161. 10.1016/j.biologicals.2007.10.002 18561375

[58] Werner GH, Schlesinger W. Morphological and quantitative comparison between infectious and non-infectious forms of influenza virus. J Exp Med. 1954;100: 203–216. 13286424 10.1084/jem.100.2.203 PMC2136367

[59] Mahy BWJ, Kangroo HO. Virology methods manual London: Academic Press, Inc; 1996.

[60] Frensing T, Kupke SY, Bachmann M, Fritzsche S, Gallo-Ramirez LE, Reichl U. Influenza virus intracellular replication dynamics, release kinetics, and particle morphology during propagation in MDCK cells. Appl Microbiol Biotechnol. 2016;100: 7181–7192. 10.1007/s00253-016-7542-4 27129532 PMC4947482

[61] Livak KJ, Schmittgen TD. Analysis of relative gene expression data using real-time quantitative PCR and the 2(-Delta Delta C(T)) Method. Methods. 2001;25: 402–8. 10.1006/meth.2001.1262 11846609

[62] Momose F, Kikuchi Y, Komase K, Morikawa Y. Visualization of microtubule-mediated transport of influenza viral progeny ribonucleoprotein. Microbes Infect. 2007;9: 1422–1433. 10.1016/j.micinf.2007.07.007 17905627

