# Supplementary material for: Model-based analysis of influenza A virus replication in genetically engineered cell lines elucidates the impact of host cell factors on key kinetic parameters of virus growth
Source: PLoS Comput Biol. 2019 Apr 11;15(4):e1006944. doi: 10.1371/journal.pcbi.1006944 (PMC6478349; doi:10.1371/journal.pcbi.1006944)
Supplement: S1 Table — (DOCX) [file pcbi.1006944.s001.docx]

**S1 Table. Summary of *in silico* optimized kinetic parameters and corresponding model response according to the analysis shown in Fig 1.**

| **Rate** | **Unit** | **Original parameter $p_{0}$** | **Optimized parameter $p_{Opt}$** | **Cell-specific virus yield $V^{Rel}(p_{Opt})$** | **$\frac{p_{Opt}}{p_{0}}$** | **$\frac{V^{Rel}(p_{Opt})}{V^{Rel}(p_{0})}$** |
| --- | --- | --- | --- | --- | --- | --- |
| **$F_{Spl8}$** | $-$ | 0.125 | 0.625 | 1.01 x 10^3^ | 5.00 | 1.04 |
| **$k_{hi}^{Att}$** | $sites^{-1}\cdot h^{-1}$ | 0.081 | 0.405 | 9.72 x 10^2^ | 5.00 | 1.00 |
| **$k^{En}$** | $h^{-1}$ | 4.800 | 23.998 | 9.74 x 10^2^ | 5.00 | 1.00 |
| **$k^{Fus}$** | $h^{-1}$ | 3.210 | 16.050 | 9.73 x 10^2^ | 5.00 | 1.00 |
| **$k^{Imp}$** | $h^{-1}$ | 0.296 | 1.480 | 9.81 x 10^2^ | 5.00 | 1.01 |
| **$k^{Exp}$** | $molecule^{-1}\cdot h^{-1}$ | 1.00 x 10^-6^ | 5.00 x 10^-6^ | 1.01 x 10^3^ | 5.00 | 1.04 |
| **$k_{P}^{Syn}$** | $nt\cdot h^{-1}$ | 6.48 x 10^4^ | 3.24 x 10^5^ | 1.27 x 10^4^ | 5.00 | 13.03 |
| **$k_{V}^{Syn}$** | $h^{-1}$ | 100.93 | 20.19 | 1.98 x 10^3^ | 0.20 | 2.04 |
| **$k_{C}^{Syn}$** | $h^{-1}$ | 1.53 | 0.31 | 2.12 x 10^3^ | 0.20 | 2.18 |
| **$k_{M}^{Syn}$** | $nt\cdot h^{-1}$ | 3.06 x 10^4^ | 1.53 x 10^5^ | 1.27 x 10^4^ | 5.00 | 13.03 |
| **$k_{RdRp}^{Bind}$** | $molecule^{-2}\cdot h^{-1}$ | 1.00 | 4.94 | 9.72 x 10^2^ | 4.94 | 1.00 |
| **$k_{NP}^{Bind}$** | $molecule^{-1}\cdot h^{-1}$ | 3.01 x 10^-4^ | 6.02 x 10^-5^ | 2.87 x 10^3^ | 0.20 | 2.95 |
| **$k_{M1}^{Bind}$** | $molecule^{-1}\cdot h^{-1}$ | 1.82 x 10^-6^ | 3.64 x 10^-7^ | 5.05 x 10^3^ | 0.20 | 5.20 |
| **$k^{Rel}$** | $virions\cdot molecule^{-1}\cdot h^{-1}$ | 1.10 x 10^-3^ | 5.50 x 10^-3^ | 4.66 x 10^3^ | 5.00 | 4.79 |
