# Supplementary material for: Model-based analysis of influenza A virus replication in genetically engineered cell lines elucidates the impact of host cell factors on key kinetic parameters of virus growth
Source: PLoS Comput Biol. 2019 Apr 11;15(4):e1006944. doi: 10.1371/journal.pcbi.1006944 (PMC6478349; doi:10.1371/journal.pcbi.1006944)
Supplement: S2 Table — (DOCX) [file pcbi.1006944.s002.docx]

**S2 Table. Uniprot identifier, names and functions of host cell genes used in this study.**

| Entry | Entry name | Protein names | Gene names | Function |
| --- | --- | --- | --- | --- |
| P40199 | CEAM6_HUMAN | Carcinoembryonic antigen-related cell adhesion molecule 6 (Non-specific cross-reacting antigen) (Normal cross-reacting antigen) (CD antigen CD66c) | CEACAM6, NCA | Direct interaction with viral NA, activates the Src/Akt survival pathway [1] |
| O15287 | FANCG_HUMAN | Fanconi anemia group G protein (Protein FACG) (DNA repair protein XRCC9) | FANCG, XRCC9 | DNA repair, direct interaction with the viral polymerase, knockdown results in reduced virus replication, overexpression increases virus replication (minigenome replicon assay) [2] |
| Q9UBU9 | NXF1_HUMAN | Nuclear RNA export factor 1 (Tip-associated protein) (Tip-associating protein) (mRNA export factor TAP) | NXF1, TAP | Inhibition of NXF1 results in less nuclear export of influenza virus mRNA for HA and NA in both HEK293T and A549 cells [3] |
| O14939 | PLD2_HUMAN | Phospholipase D2 (PLD 2) (hPLD2) (EC 3.1.4.4) (Choline phosphatase 2) (PLD1C) (Phosphatidylcholine-hydrolyzing phospholipase D2) | PLD2 | RNAi of PLD delays influenza virus entry and reduced viral titers *in vitro*; *in vivo*, PLD2 inhibition reduces virus titer and correlates with significant increase in transcription of innate antiviral effectors [4] |
| Q9HCS7 | SYF1_HUMAN | Pre-mRNA-splicing factor SYF1 (Protein HCNP) (XPA-binding protein 2) | XAB2, HCNP, KIAA1177, SYF1, PP3898 | DNA repair mechanism, transcription and transcription-coupled repair [5], host restriction factor for various viruses [6]) |

Supporting information references

1. Gaur P, Ranjan P, Sharma S, Patel JR, Bowzard JB, Rahman SK, et al. Influenza A virus neuraminidase protein enhances cell survival through interaction with carcinoembryonic antigen-related cell adhesion molecule 6 (CEACAM6) protein. J Biol Chem. 2012;287: 15109–15117. doi:10.1074/jbc.M111.328070

2. Tafforeau L, Chantier T, Pradezynski F, Pellet J, Mangeot PE, Vidalain P, et al. Generation and comprehensive analysis of an influenza virus polymerase cellular interaction network. J Virol. 2011;85: 13010–8. doi:10.1128/JVI.02651-10

3. Larsen S, Bui S, Perez V, Mohammad A, Medina-Ramirez H, Newcomb LL. Influenza polymerase encoding mRNAs utilize atypical mRNA nuclear export. Virol J. 2014;11: 1–11. doi:10.1186/1743-422X-11-154

4. Oguin TH, Sharma S, Stuart AD, Duan S, Scott SA, Jones CK, et al. Phospholipase D facilitates efficient entry of influenza virus, allowing escape from innate immune inhibition. J Biol Chem. 2014;289: 25405–25417. doi:10.1074/jbc.M114.558817

5. Kuraoka I, Ito S, Wada T, Hayashida M, Lee L, Saijo M, et al. Isolation of XAB2 complex involved in pre-mRNA splicing, transcription, and transcription-coupled repair. J Biol Chem. 2008;283: 940–950. doi:10.1074/jbc.M706647200

6. Thakur A, Qureshi A, Kumar M. VhfRNAi: A web-platform for analysis of host genes involved in viral infections discovered by genome wide RNAi screens. Mol Biosyst. Royal Society of Chemistry; 2017;13: 1377–1387. doi:10.1039/c6mb00841k
