# Supplementary material for: Model-based analysis of influenza A virus replication in genetically engineered cell lines elucidates the impact of host cell factors on key kinetic parameters of virus growth
Source: PLoS Comput Biol. 2019 Apr 11;15(4):e1006944. doi: 10.1371/journal.pcbi.1006944 (PMC6478349; doi:10.1371/journal.pcbi.1006944)
Supplement: S3 Table — (DOCX) [file pcbi.1006944.s003.docx]

**S3 Table. Overexpression level of host cell genes in cell lines overexpressing one of the indicated genes as determined by $2^{-\Delta\Delta C_{T}}$ method.**

| **Gene overexpressed in corresponding SGOs** | **Fold overexpression compared to parental A549 cells** |
| --- | --- |
| **CEACAM6** | 19 |
| **FANCG** | 7 |
| **NXF1** | 1.5 |
| **PLD2** | 14 |
| **XAB2** | 3 |
