# Supplementary material for: Model-based analysis of influenza A virus replication in genetically engineered cell lines elucidates the impact of host cell factors on key kinetic parameters of virus growth
Source: PLoS Comput Biol. 2019 Apr 11;15(4):e1006944. doi: 10.1371/journal.pcbi.1006944 (PMC6478349; doi:10.1371/journal.pcbi.1006944)
Supplement: S4 Table — (DOCX) [file pcbi.1006944.s004.docx]

**S4 Table. Overexpression level of host cell genes in cell lines overexpressing multiple genes (MGOs) as determined by $2^{-\Delta\Delta C_{T}}$ method.**

| **Cell line →** | **control** | **MGO 1** | **MGO 2** | **MGO 3** | **MGO 4** |
| --- | --- | --- | --- | --- | --- |
| **Gene name ↓** | **Fold overexpression compared to parental A549 cells** | | | | |
| **CEACAM6** | 3 | 12 | 57 | 25 | - |
| **FANCG** | 1 | 10 | 1 | 20 | 15 |
| **NXF1** | 1 | 2 | 3 | 2 | - |
| **PLD2** | 1 | 1 | 1 | 2.5 | 4 |
| **XAB2** | 1 | 1.5 | 7 | 4 | 11 |

“-“ Gene was not overexpressed; strength of overexpression levels is visualized by color shadings with respect to each gene indicated on the left-hand side of each row of the table.
