# Supplementary material for: Model-based analysis of influenza A virus replication in genetically engineered cell lines elucidates the impact of host cell factors on key kinetic parameters of virus growth
Source: PLoS Comput Biol. 2019 Apr 11;15(4):e1006944. doi: 10.1371/journal.pcbi.1006944 (PMC6478349; doi:10.1371/journal.pcbi.1006944)
Supplement: S5 Table — (DOCX) [file pcbi.1006944.s005.docx]

**S5 Table. Comparison of key kinetic parameters of influenza A virus replication in parental A549 cells and A549 cells overexpressing selected host cell factors (SGOs).**

| **Parameter →** | **$k^{Imp}$** | **$k_{V}^{Syn}$** | **$k_{C}^{Syn}$** | **$k_{M}^{Syn}$** | **$k_{M1}^{Bind}$** | **$k^{Rel}$** |
| --- | --- | --- | --- | --- | --- | --- |
| **Description →** | Nuclear vRNP import | vRNA synthesis | cRNA synthesis | mRNA synthesis | Binding of M1 to nuclear vRNPs | Virus release |
| **Unit →** | $h^{-1}$ | $h^{-1}$ | $h^{-1}$ | $nt\cdot h^{-1}$ | $molecule^{-1}\cdot h^{-1}$ | $virions\cdot molecule^{-1}\cdot h^{-1}$ |
| **Gene name ↓** | Parameter values **↓** | | | | | |
| **A549 cells** | 0.296 | 100.93 | 1.53 | 3.06 x 10^4^ | 1.82 x 10^-6^ | 1.10 x 10^-3^ |
| **control** | 0.281 | 93.66 | 1.48 | 3.62 x 10^4^ | 2.14 x 10^-6^ | 0.98 x 10^-3^ |
| **CEACAM6*** | 0.199 | 142.56 | 1.65 | 2.34 x 10^4^ | 1.73 x 10^-6^ | 2.20 x 10^-3^ |
| **FANCG** | 0.258 | 137.45 | 0.88 | 1.91 x 10^4^ | 1.81 x 10^-6^ | 2.60 x 10^-3^ |
| **NXF1** | 0.209 | 151.89 | 1.48 | 1.97 x 10^4^ | 1.34 x 10^-6^ | 3.10 x 10^-3^ |
| **PLD2** | 0.287 | 156.26 | 0.80 | 2.15 x 10^4^ | 1.54 x 10^-6^ | 1.70 x 10^-3^ |
| **XAB2** | 0.295 | 95.07 | 1.10 | 2.71 x 10^4^ | 1.58 x 10^-6^ | 1.20 x 10^-3^ |

*Name of the host cell gene that was overexpressed in the corresponding SGO.
