# Supplementary material for: Model-based analysis of influenza A virus replication in genetically engineered cell lines elucidates the impact of host cell factors on key kinetic parameters of virus growth
Source: PLoS Comput Biol. 2019 Apr 11;15(4):e1006944. doi: 10.1371/journal.pcbi.1006944 (PMC6478349; doi:10.1371/journal.pcbi.1006944)
Supplement: S6 Table — (DOCX) [file pcbi.1006944.s006.docx]

**S6 Table. Parameters used for the simulation of intracellular IAV replication.**

| **Parameter** | | **Description** | **Value** | **Unit** | **Source, comment** |
| --- | --- | --- | --- | --- | --- |
| **$B_{hi}^{tot}$** | number of  high-affinity binding sites | | 150 | *sites* | [1] |
| **$B_{lo}^{tot}$** | number of  low-affinity binding sites | | 1000 | *sites* | [1] |
| **$D_{Rib}$** | distance between two adjacent ribosomes | | 160 | *nucleotides* | [2] |
| **$F_{Fus}$** | fraction of  fusion-competent virions | | 0.51 | *–* | [3] |
| **$F_{Fspl7}$** | fraction of  M2-encoding mRNAs | | 0.02 | *–* | based on ratio of M2 to M1 in a virion |
| **$F_{Fspl8}$** | fraction of  NEP-encoding mRNAs | | 0.125 | *–* | [4] |
| **$k_{hi}^{Att}$** | attachment to high-affinity binding sites | | 8.09 x 10^-2^ | *sites^-1^∙h^-1^* | adjusted to data in [1] |
| **$k_{lo}^{Att}$** | attachment to low-affinity binding sites | | 4.55 x 10^-4^ | *sites^-1^∙h^-1^* | adjusted to data in [1] |
| **$k_{NP}^{Bind}$** | binding of NP to  RdRp-RNA complexes | | 3.01 x 10^-4^ | *molecule^-1^∙h^-1^* | [3] |
| **$k_{RdRp}^{Bind}$** | binding of RdRp-complexes  to RNA | | 1 | *molecule^-1^∙h^-1^* | [3] |
| **$k_{M}^{Deg}$** | degradation of mRNA | | 0.33 | *h^-1^* | [3] |
| **$k_{R}^{Deg}$** | degradation of nascent cRNA/vRNA | | 36.36 | *h^-1^* | [3] |
| **$k_{Rnp}^{Deg}$** | degradation of RNPs | | 0.09 | *h^-1^* | [3] |
| **$k_{RRdRp}^{Deg}$** | degradation of RdRp-RNA complexes | | 4.25 | *h^-1^* | [3] |
| **$k^{En}$** | endocytosis | | 4.8 | *h^-1^* | [3] |
| **$k_{hi}^{Eq}$** | equilibrium constant of high-affinity sites | | 1.13 x 10^-2^ | *sites^-1^* | [1] |
| **$k_{lo}^{Eq}$** | equilibrium constant of low-affinity sites | | 8.33 x 10^-5^ | *sites^-1^* | [1] |
| **$k^{Exp}$** | NEP binding and nuclear export | | 1.00 x 10^-6^ | *molecule^-1^∙h^-1^* | adjusted to [5] |
| **$k^{Fus}$** | fusion with endosomes | | 3.21 | *h^-1^* | [3] |
| **$k^{RdRp}$** | formation of RdRp-complexes | | 1 | *molecule^-2^∙h^-1^* | rapid formation is assumed |
| **$k_{P}^{Syn}$** | protein synthesis | | 64800 | *nucleotides∙h^-1^* | [6] |
| **$K_{V^{Rel}}$** | influence of proteins on virus release | | 10 | *virions* | [3] |
| **$L_{1}$** | length of segment 1’s mRNA | | 2320 | *nucleotides* | [7] |
| **$L_{2}$** | length of segment 2’s mRNA | | 2320 | *nucleotides* | [7] |
| **$L_{3}$** | length of segment 3’s mRNA | | 2211 | *nucleotides* | [7] |
| **$L_{4}$** | length of segment 4’s mRNA | | 1757 | *nucleotides* | [7] |
| **$L_{5}$** | length of segment 5’s mRNA | | 1540 | *nucleotides* | [7] |
| **$L_{6}$** | length of segment 6’s mRNA | | 1392 | *nucleotides* | [7] |
| **$L_{7}$** | length of segment 7’s unspliced mRNA | | 1005 | *nucleotides* | [7] |
| **$L_{8}$** | length of segment 8’s unspliced mRNA | | 868 | *nucleotides* | [7] |
| **$L^{V}$** | average length of a vRNA | | 1700 | *nucleotides* | based on [7] |
| **$N_{\text{P}_{\text{RdRp}}}$** | Number of RdRP-complexes  in a virion | | 45 | *molecules∙virion^-1^* | [7] |
| **$N_{\text{P}_{\text{HA}}}$** | number of HA molecules in a virion | | 500 | *molecules∙virion^-1^* | [7] |
| **$N_{\text{P}_{\text{NA}}}$** | number of NA molecules in a virion | | 100 | *molecules∙virion^-1^* | [7] |
| **$N_{\text{P}_{\text{NP}}}$** | Number of NP molecules  in a virion | | 1000 | *molecules∙virion^-1^* | [7] |
| **$N_{\text{P}_{M1}}$** | number of M1 molecules in a virion | | 3000 | *molecules∙virion^-1^* | [7] |
| **$N_{\text{P}_{M2}}$** | number of M2 molecules in a virion | | 40 | *molecules∙virion^-1^* | [7] |
| **$N_{\text{P}_{\text{NEP}}}$** | number of NEP molecules  in a virion | | 165 | *molecules∙virion^-1^* | [7] |
| **$N_{M1}^{Nuc}$** | nucleotides bound by one M1 molecule | | 200 | *nucleotides* | [8] |
| **$N_{NP}^{Nuc}$** | nucleotides bound by one NP molecule | | 24 | *nucleotides* | [9] |

Supporting information references

1. Nunes-Correia I, Ramalho-Santos J, Nir S, Pedroso de Lima MC. Interactions of Influenza Virus with Cultured Cells: Detailed Kinetic Modeling of Binding and Endocytosis. Biochemistry. 1999;38: 1095–1101. doi:10.1021/bi9812524

2. Arava Y, Wang Y, Storey JD, Liu CL, Brown PO, Herschlag D. Genome-wide analysis of mRNA translation profiles in Saccharomyces cerevisiae. Proc Natl Acad Sci U S A. 2003;100: 3889–94. doi:10.1073/pnas.0635171100

3. Heldt FS, Frensing T, Reichl U. Modeling the Intracellular Dynamics of Influenza Virus Replication To Understand the Control of Viral RNA Synthesis. Journal of Virology. 2012. pp. 7806–7817. doi:10.1128/JVI.00080-12

4. Robb NC, Jackson D, Vreede FT, Fodor E. Splicing of influenza A virus NS1 mRNA is independent of the viral NS1 protein. J Gen Virol. 2010;91: 2331–40. doi:10.1099/vir.0.022004-0

5. Amorim MJ, Bruce EA, Read EKC, Foeglein A, Mahen R, Stuart AD, et al. A Rab11- and microtubule-dependent mechanism for cytoplasmic transport of influenza A virus viral RNA. J Virol. 2011;85: 4143–56. doi:10.1128/JVI.02606-10

6. Spirin AS. Ribosome structure and protein biosynthesis. Menlo Park, CA: Benjamin/Cummings Pub. Co.; 1986.

7. Lamb RA, Krug RM. Orthomyxoviridae: the viruses and their replication. In: Knipe DM, Howley PM, editors. Fields Virology. 4th ed. Philadelphia, Pa: Lippincott Williams & Wilkins; 2001. pp. 1487–1531.

8. Wakefield L, Brownlee GG. RNA-binding properties of influenza A virus matrix protein M1. Nucleic Acids Res. 1989;17: 8569–80.

9. Portela A, Digard P. The influenza virus nucleoprotein: a multifunctional RNA-binding protein pivotal to virus replication. J Gen Virol. 2002;83: 723–34.
