# Supplementary material for: Model-based analysis of influenza A virus replication in genetically engineered cell lines elucidates the impact of host cell factors on key kinetic parameters of virus growth
Source: PLoS Comput Biol. 2019 Apr 11;15(4):e1006944. doi: 10.1371/journal.pcbi.1006944 (PMC6478349; doi:10.1371/journal.pcbi.1006944)
Supplement: S7 Table — (DOCX) [file pcbi.1006944.s007.docx]

**S7 Table. Primer sets for reverse transcription and real-time RT-qPCR for segment 5 of A/PR/8/34 (H1N1).**

| **Target** | **Purpose** | **Primer Name** | **Sequence (5’-3’)** | **Position (nt)** |
| --- | --- | --- | --- | --- |
| **mRNA** | Reverse transcription | Oligo tagdTRT rev | GTAAAACGACGGCCAGTTTTTTTTTTTTTTTTT | polyA tail |
|  | Real-time RT-qPCR | Seg 5 Realtime for | GGAAAGTGCAAGACCAGAAGAT | 1388 - 1410 |
|  | Real-time RT-qPCR | mRNA tagRealtime rev | GTAAAACGACGGCCAGT | Tag seq. |
| **cRNA** | Reverse transcription | Seg 5 tagRT N rev | GCTAGCTTCAGCTAGGCATCAGTAGAAACAAGGGTATTTTTCTT | 1541 - 1565 |
|  | Real-time RT-qPCR | Seg 5 Realtime for | GGAAAGTGCAAGACCAGAAGAT | 1388 - 1410 |
|  | Real-time RT-qPCR | cRNA tagRealtime rev | GCTAGCTTCAGCTAGGCATC | Tag seq. |
| **vRNA** | Reverse transcription | Seg 5 tagRT SP6 for | ATTTAGGTGACACTATAGAAGCGAGTGATTATGAGGGACGGTTGAT | 192 - 215 |
|  | Real-time RT-qPCR | Seg 5 Realtime rev | CGCACTGGGATGTTCTTC | 282 – 300 |
|  | Real-time RT-qPCR | vRNA tagRealtime for | ATTTAGGTGACACTATAGAAGCG | Tag seq. |
