# Supplementary material for: Model-based analysis of influenza A virus replication in genetically engineered cell lines elucidates the impact of host cell factors on key kinetic parameters of virus growth
Source: PLoS Comput Biol. 2019 Apr 11;15(4):e1006944. doi: 10.1371/journal.pcbi.1006944 (PMC6478349; doi:10.1371/journal.pcbi.1006944)
Supplement: S8 Table — (DOCX) [file pcbi.1006944.s008.docx]

**S8 Table. Primer sets for the generation of RNA reference standards for A/PR/8/34 (H1N1) segment 5.**

| **RNA species** | **Primer name** | **Sequence (5’–3’)** | **Position (nt)** |
| --- | --- | --- | --- |
| **mRNA** | Seg 5 Uni T7 for | TAATACGACTCACTATAGGGAGCAAAAGCAGGGTAGATAATC | 1 – 22 |
|  | Seg 5 dT rev | TTTTTTTTTTTTTTTTCTTTAATTGTC | 1533 – 1549 |
| **cRNA** | Seg 5 Uni T7 for | TAATACGACTCACTATAGGGAGCAAAAGCAGGGTAGATAATC | 1 – 22 |
|  | Seg 5 Uni rev | AGTAGAAACAAGGGTATTTTTC | 1543 – 1565 |
| **vRNA** | Seg 5 Uni for | AGCAAAAGCAGGGTAGATAATC | 1 – 22 |
|  | Seg 5 Uni T7 rev | TAATACGACTCACTATAGGGAGTAGAAACAAGGGTATTTTTC | 1543 – 1565 |
