# Supplementary material for: Model-based analysis of influenza A virus replication in genetically engineered cell lines elucidates the impact of host cell factors on key kinetic parameters of virus growth
Source: PLoS Comput Biol. 2019 Apr 11;15(4):e1006944. doi: 10.1371/journal.pcbi.1006944 (PMC6478349; doi:10.1371/journal.pcbi.1006944)
Supplement: S9 Table — (DOCX) [file pcbi.1006944.s009.docx]

**S9 Table. Primer sets for PCR of host cell mRNA.**

| **Gene name** | **Primer Name** | **Sequence (5’-3’)** |
| --- | --- | --- |
| **CEACAM6** | For | GCTCTTTATCCCCAACATCACTGT |
|  | Rev | GAGAGGACAGGAGCACTTCCAG |
| **FANCG** | For | GGTGCCCAAAAAGTGGCAAT |
|  | Rev | GCTCACAGTTGAAAGCTGCC |
| **NXF1** | For | TCAAAGGCCTCCGTTCAGAC |
|  | Rev | TGCGATTCAGGACAACGTCA |
| **PLD2** | For | TCAATGACCGGAGCTTGCTG |
|  | Rev | AGTGCTTCCGCAGACTCAAG |
| **XAB2** | For | TCCGACATCTGGAGCACCTA |
|  | Rev | AGCAGGTACAAGGTCTTGGC |
