# Supplementary figures and images for: Model-based analysis of influenza A virus replication in genetically engineered cell lines elucidates the impact of host cell factors on key kinetic parameters of virus growth
Source: PLoS Comput Biol. 2019 Apr 11;15(4):e1006944. doi: 10.1371/journal.pcbi.1006944 (PMC6478349; doi:10.1371/journal.pcbi.1006944)

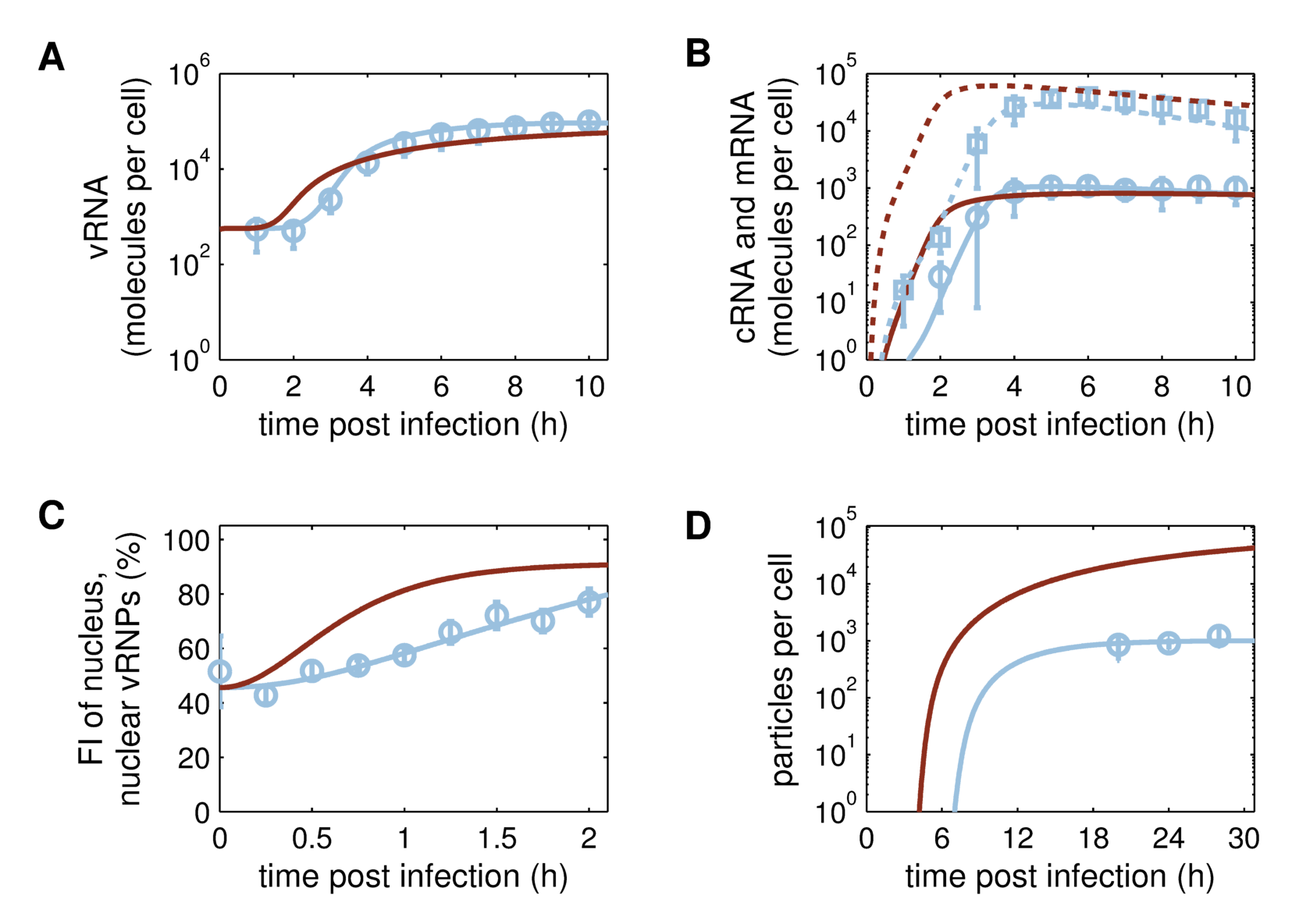

Supplement: S1 Fig — Model fit (blue lines) to experimental data (blue symbols) for A549 and simulations for MDCK cells (brown lines) are shown, respectively. (A, B) Intracellular dynamics of viral RNA for a simulated infection at MOI 50 for vRNA and cRNA (circles, solid line) as well as for mRNA (squares, dashed line) in A549 cells and MDCK cells. (C) Nuclear import of viral genomes in CHX-treated cells for a simulated infection at MOI 50. For better comparison, the simulated fraction of nuclear vRNPs in MDCK cells was compressed with respect to the vRNP offset of A549 cells. (D) Cell-specific virus release for a simulated infection at MOI 1. (TIF) [file pcbi.1006944.s010.tif]

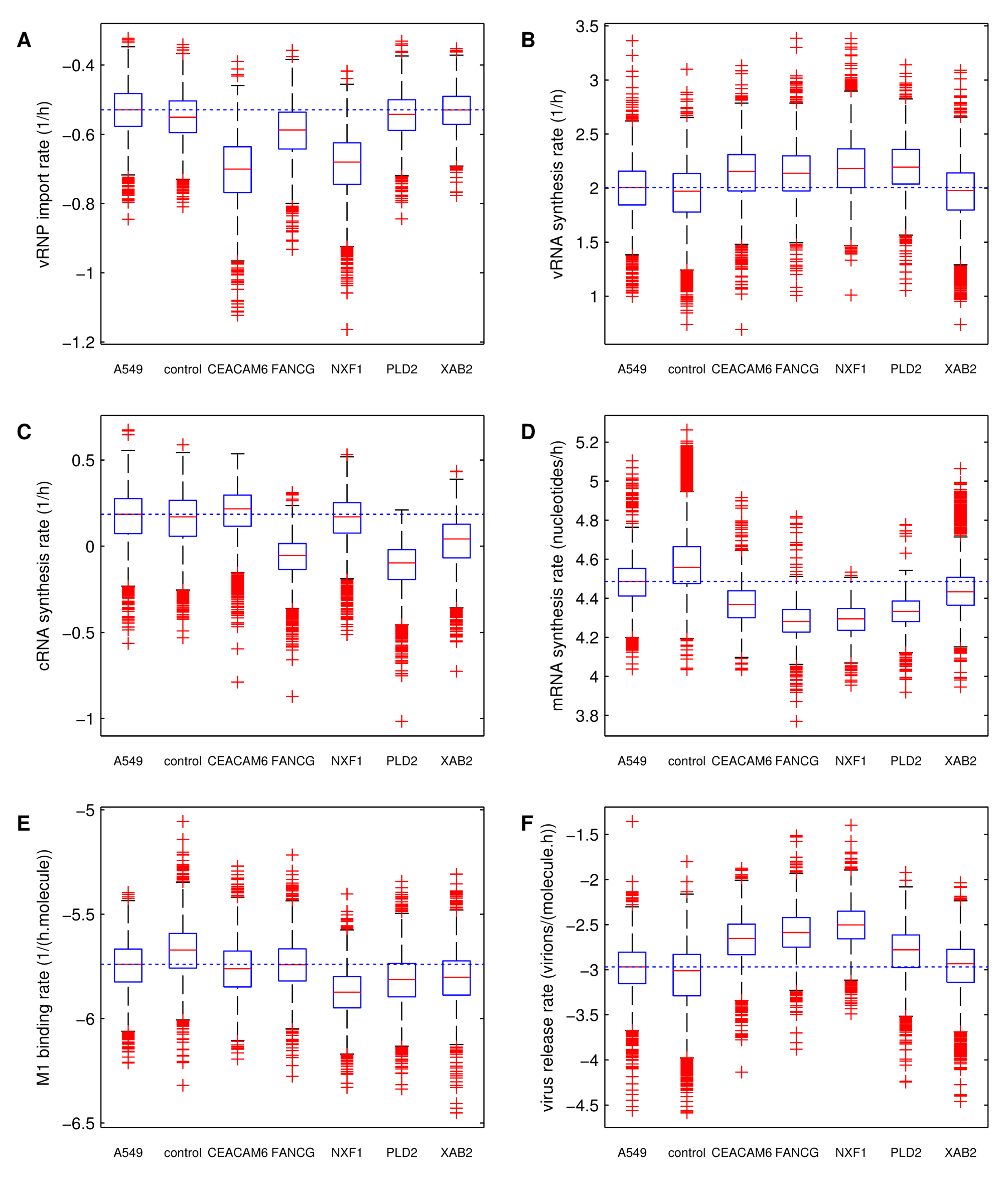

Supplement: S2 Fig — Decadic logarithm of parameter values for fitting 3000 resamplings of the available experimental data obtained from SGOs. Shown are median (red solid line), first and third quartile (blue box), maximum values (whiskers) and outliers (red crosses). Blue dashed lines represent the median of the respective parameter in parental A549 cells. Experimental data for estimating the nuclear vRNP import rate in cycloheximide-treated cells (A) were resampled separately from those used for simultaneous estimation of vRNA (B), cRNA (C), mRNA (D), M1 binding (E) and virus release rate (F) in conventional infection experiments (without CHX treatment). (TIF) [file pcbi.1006944.s011.tif]

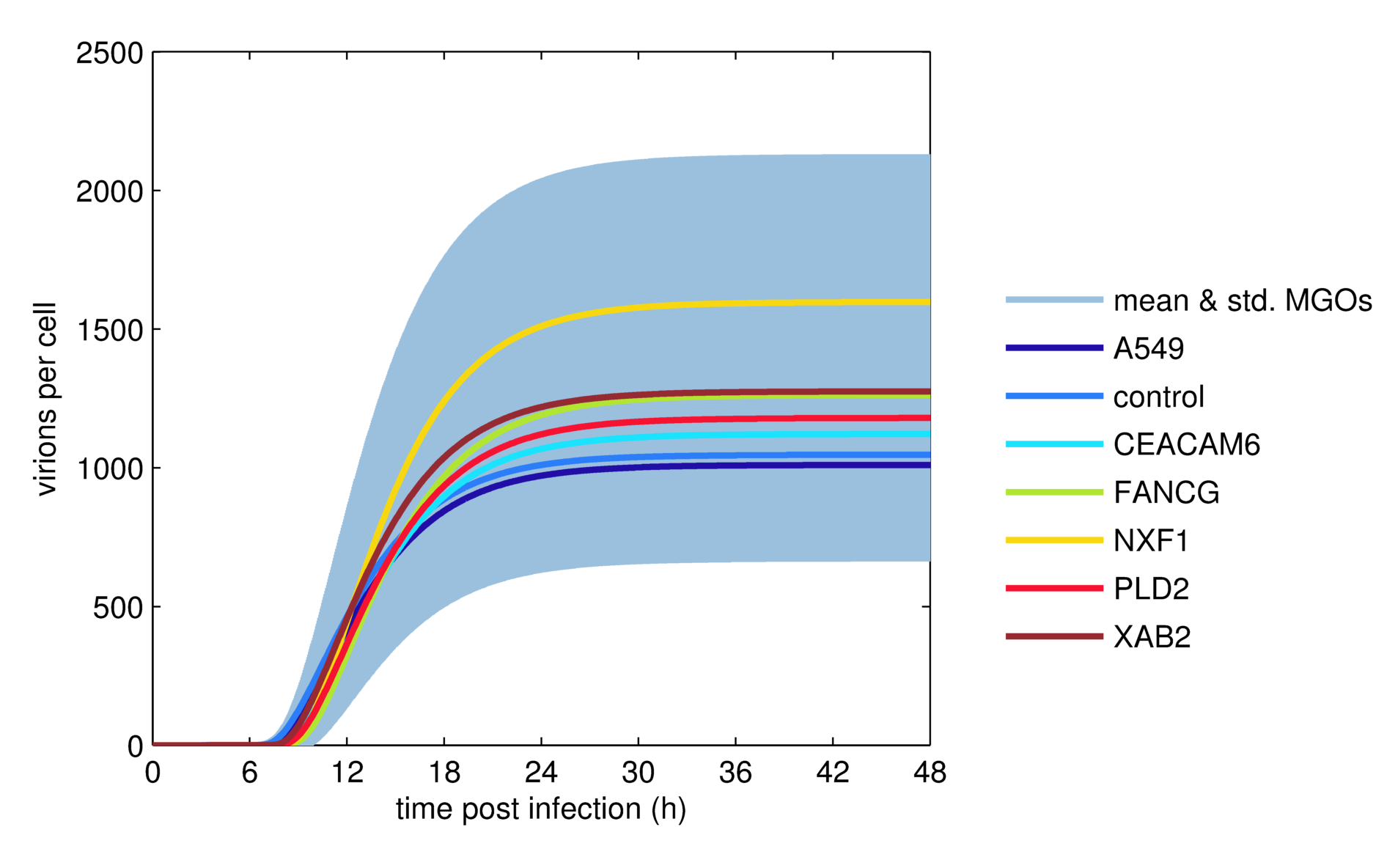

Supplement: S3 Fig — Light blue area shows the mean and standard deviation of released virions from approximately 2 x 10^4^ simulations with randomized parameter sets, for a simulated infection at MOI 1. Infection of parental A549 cells, the transduction control and SGOs were simulated with the optimized parameter sets as determined in the present study (colors according to legend). (TIF) [file pcbi.1006944.s012.tif]

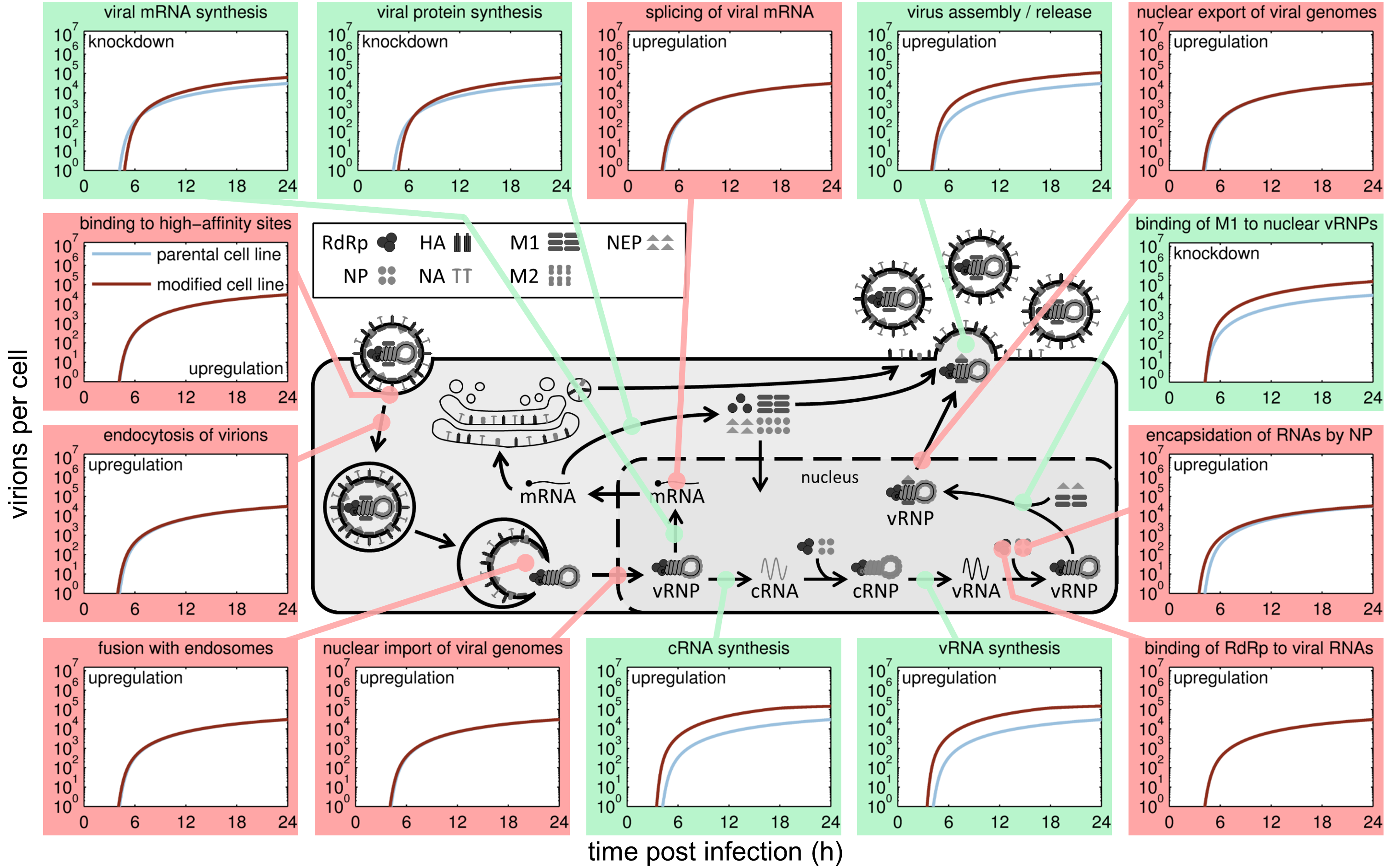

Supplement: S4 Fig — We assume that efficiency of individual steps in the virus life cycle is directly dependent on host cell factors and that their influence is changed upon knockdown or overexpression of the corresponding gene. We simulated manipulation of gene expression by perturbing the corresponding kinetic parameters in the IAV replication model for MDCK cells established previously by our group [12] according to the approach presented for A549 cells (Fig 1). For the most important steps, virus release of parental MDCK cells (blue solid line) and the engineered cell line (brown solid line) are shown for a simulated infection at MOI 1. Colors indicate whether perturbation of the indicated step improved final virus yield at 24 h p.i. by at least two-fold (green), or had no impact (red). Scheme of IAV replication adapted from [22]. (TIF) [file pcbi.1006944.s013.tif]

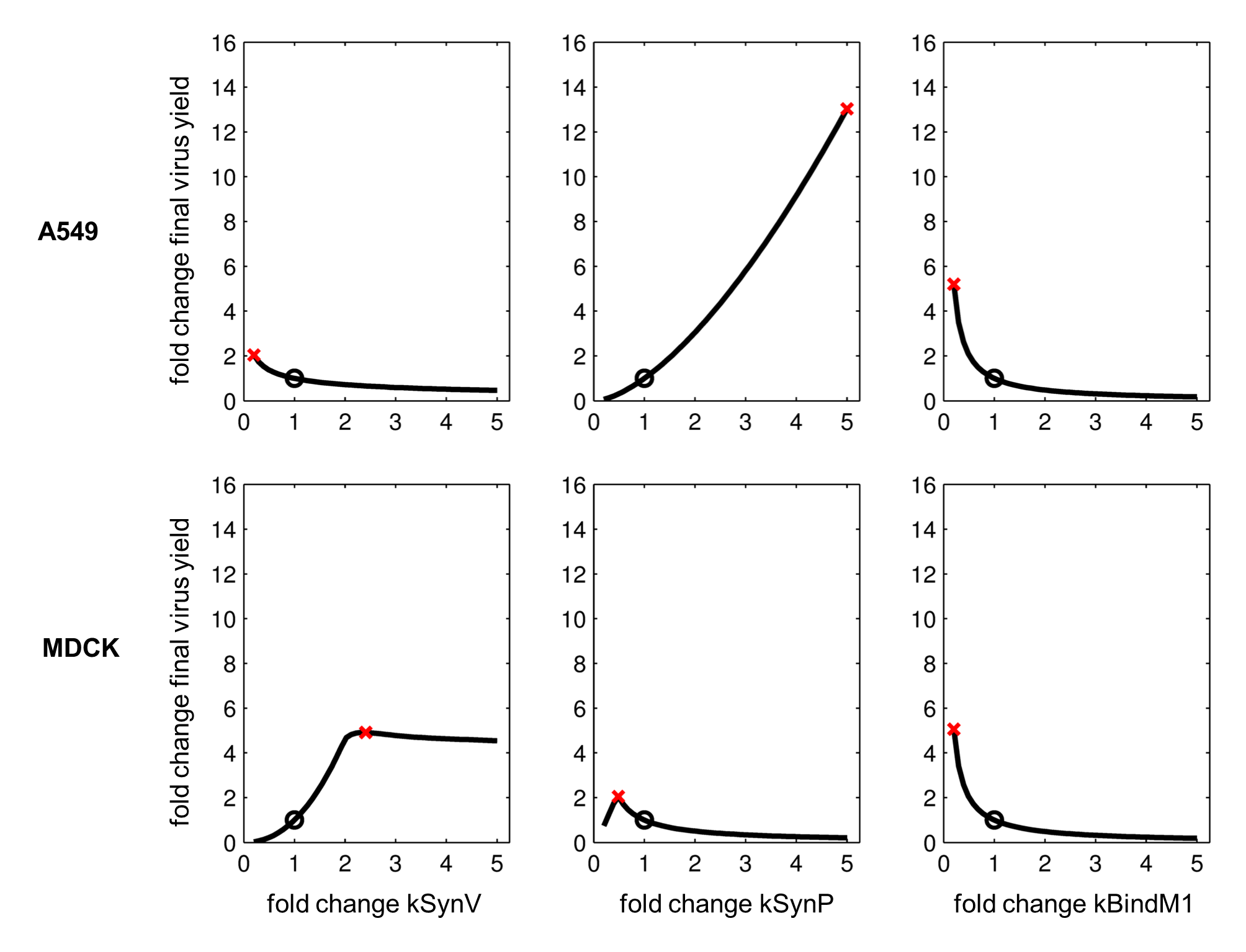

Supplement: S5 Fig — We simulated manipulation of vRNA synthesis (column 1), viral protein synthesis (column 2) and the binding of the matrix protein 1 (M1) to nuclear vRNPs (column 3) by perturbing the corresponding kinetic parameters in the IAV replication model for both A549 cells established in the present study (upper panel) and for MDCK cells established previously by our group [12] (lower panel). Shown are the fold changes of the virus yield at 24 h p.i. in response to the fold changes in the corresponding parameters (black solid lines) with respect to the simulation of the parental cell lines. For every parameter analysis the simulation read out for the parental cell line (black open circle) and the optimal cell line (red cross) is marked. (TIF) [file pcbi.1006944.s014.tif]

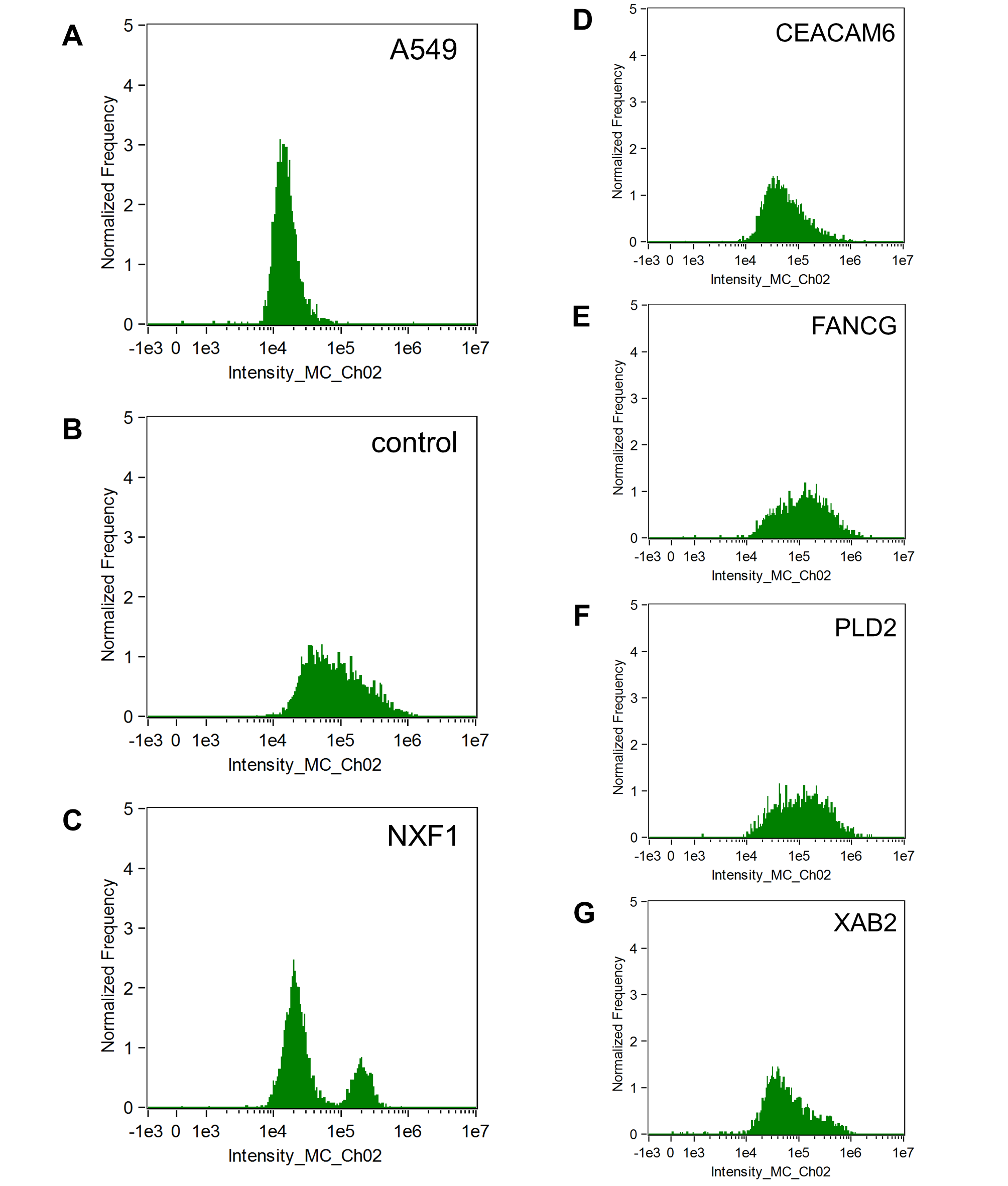

Supplement: S6 Fig — PFA-fixated cells were measured by imaging flow cytometry using the 488 nm laser. The eGFP signal of single cells in focus was evaluated using the mean FI (mean pixel feature) of channel 2 (CH02) and visualized as histograms for parental A549 cells (A), the transduction control (B) and A549 cells overexpressing one of the following host cell factors: NXF1 (C), CEACAM6 (D), FANCG (E), PLD2 (F), XAB2 (G). (TIF) [file pcbi.1006944.s015.tif]

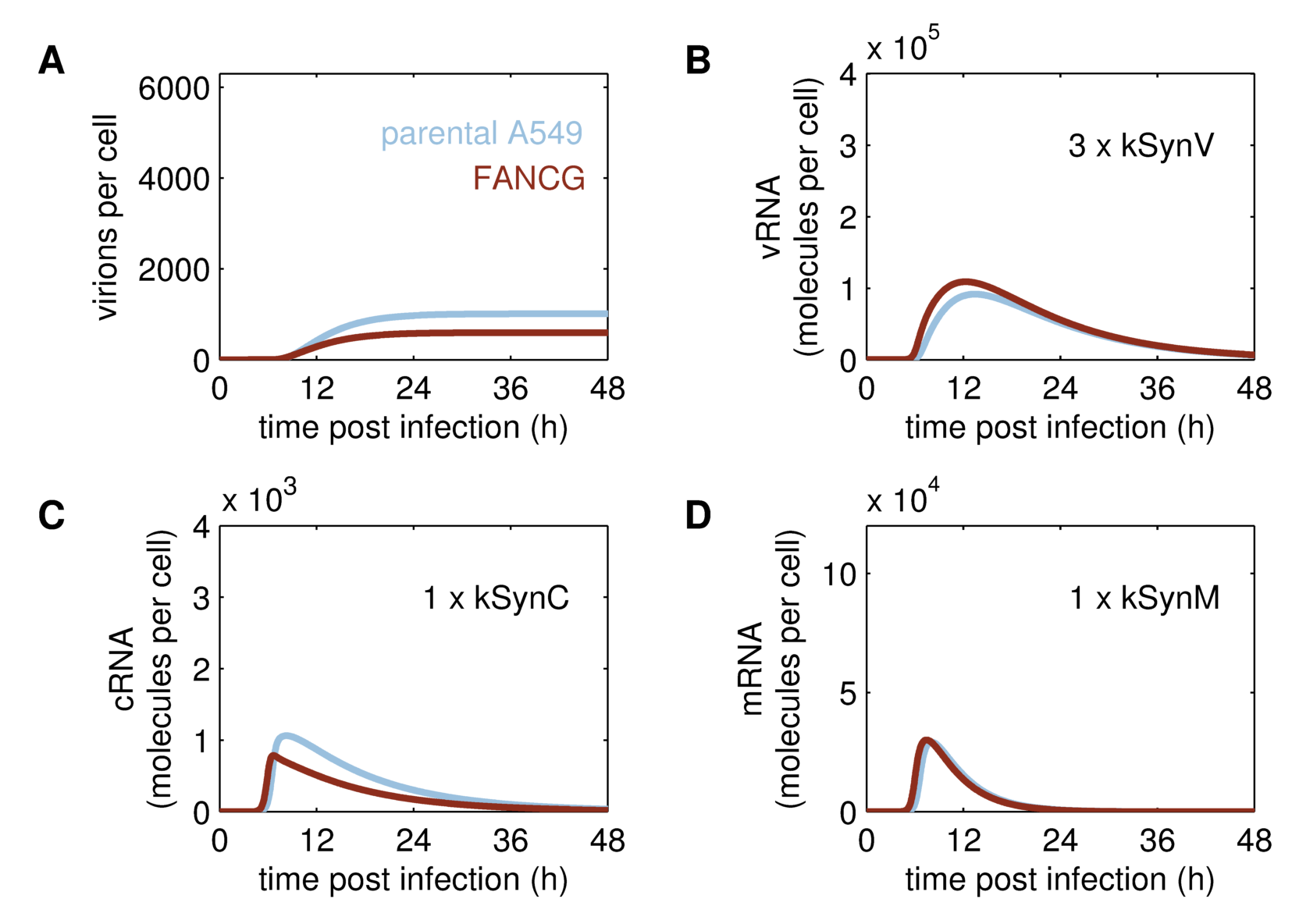

Supplement: S7 Fig — Virus particle release (A) and dynamics of intracellular vRNA (B), cRNA (C) and mRNA (D) if overexpression of FANCG causes a three-fold increase in the vRNA synthesis rate. (TIF) [file pcbi.1006944.s016.tif]

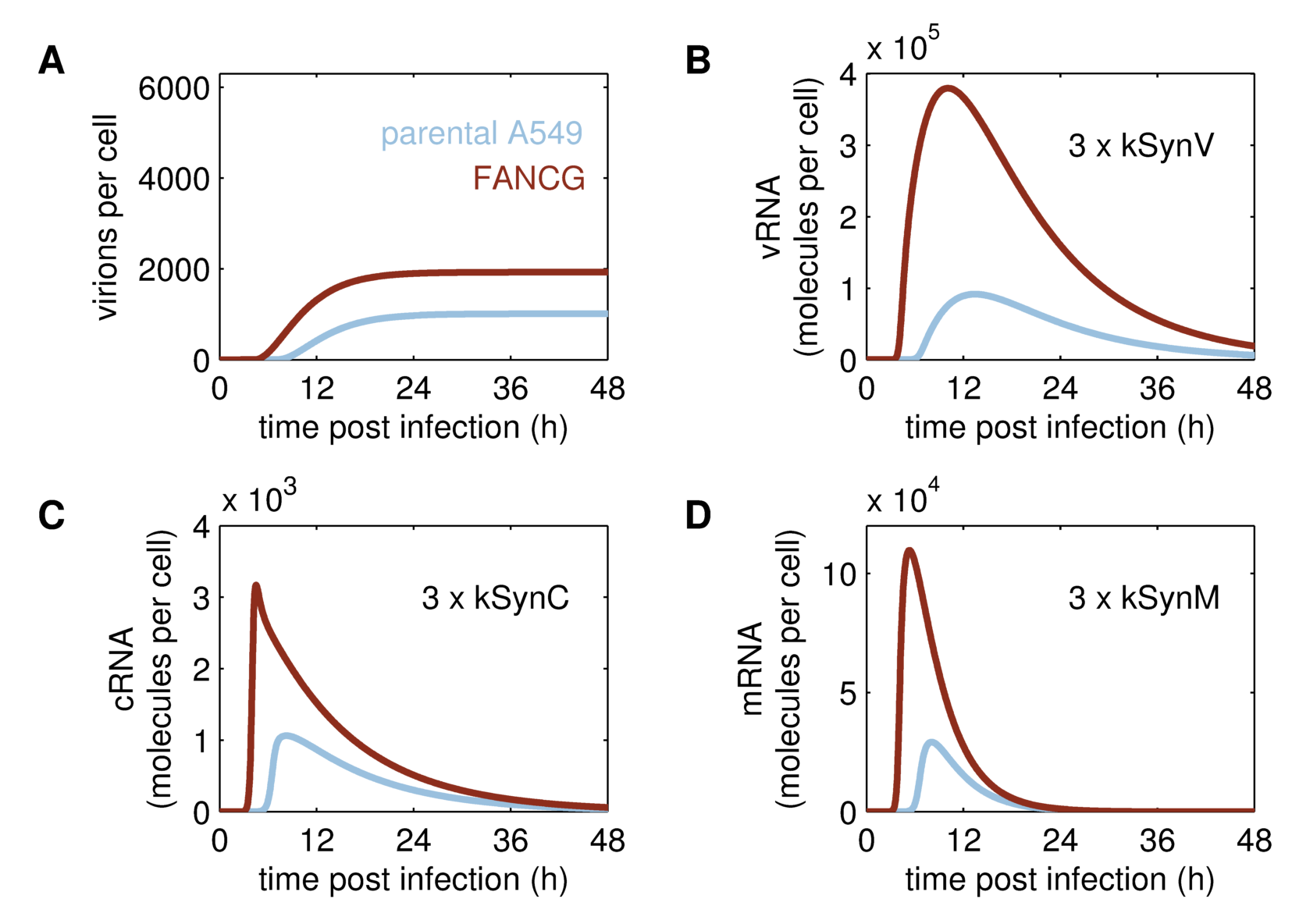

Supplement: S8 Fig — Virus particle release (A) and dynamics of intracellular vRNA (B), cRNA (C) and mRNA (D) if overexpression of FANCG causes a three-fold increase in the synthesis rates for viral vRNA, cRNA and mRNA. (TIF) [file pcbi.1006944.s017.tif]

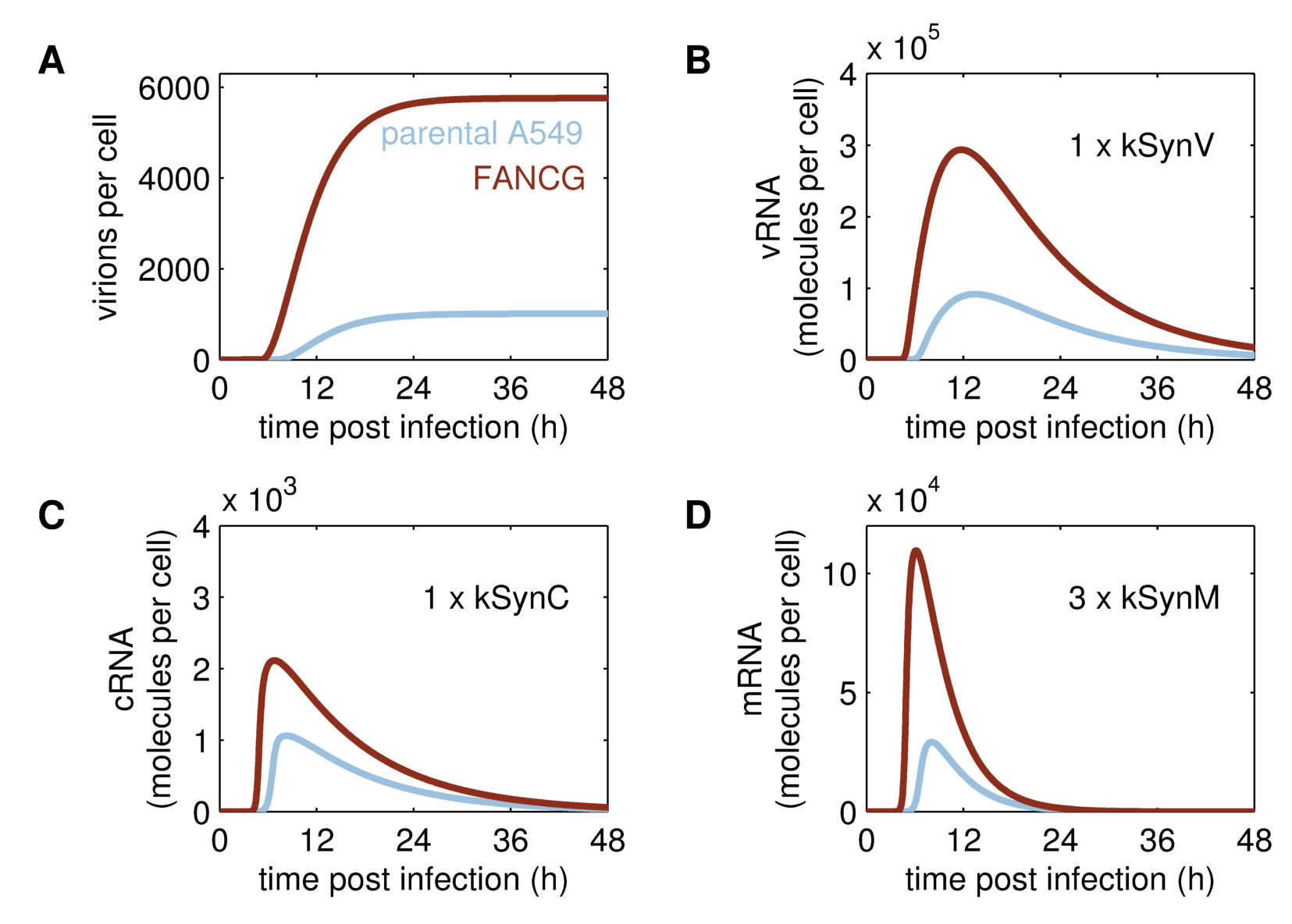

Supplement: S9 Fig — Virus particle release (A) and dynamics of intracellular vRNA (B), cRNA (C) and mRNA (D) if overexpression of FANCG causes a three-fold increase in the mRNA synthesis rates. (TIF) [file pcbi.1006944.s018.tif]

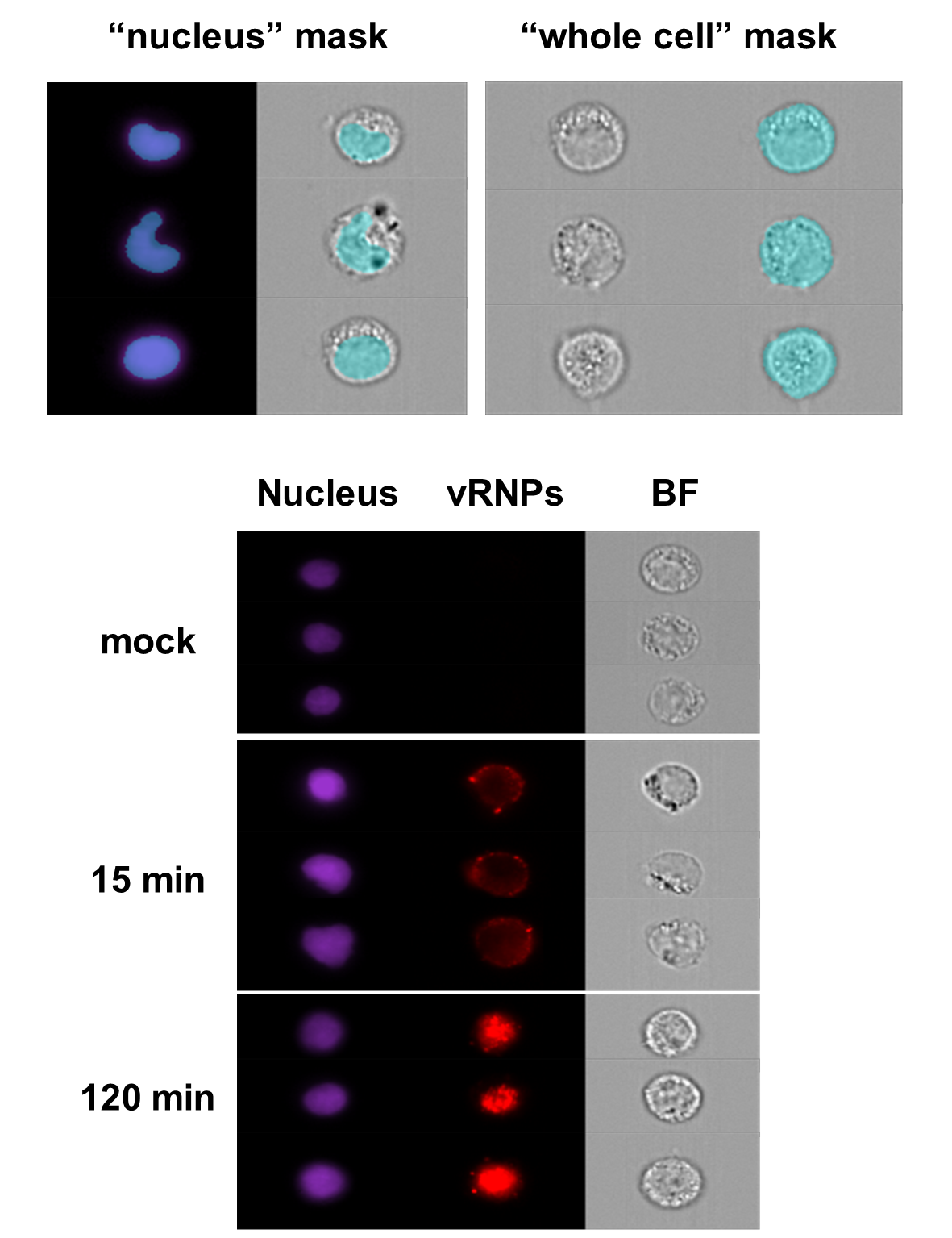

Supplement: S10 Fig — The nucleus mask was defined with the help of the “morphology” feature on CH1 (DAPI signal) and the whole cell mask with the “object” feature on CH6 (bright field). (TIF) [file pcbi.1006944.s019.tif]
