# Supplementary material for: Model-based analysis of influenza A virus replication in genetically engineered cell lines elucidates the impact of host cell factors on key kinetic parameters of virus growth
Source: PLoS Comput Biol. 2019 Apr 11;15(4):e1006944. doi: 10.1371/journal.pcbi.1006944 (PMC6478349; doi:10.1371/journal.pcbi.1006944)
Supplement: S1 File — (DOCX) [file pcbi.1006944.s020.docx]

S1 File. Mathematical model of intracellular influenza A virus replication

In the following section, we list the equations of the ODE model used in the present study to simulate IAV replication in a single cell. This model is identical to a model that was already published by our group. For a detailed description, the reader is referred to the original publication [1].

Virus entry

The binding of extracellular virus particles $V^{Ex}$ to binding sites of high affinity $B_{Hi}$ and low affinity $B_{Lo}$ is described by the following equations.

$\frac{\text{d}V^{\text{Ex}}}{\text{d}t}=k_{\text{Hi}}^{\text{Dis}}V_{\text{Hi}}^{\text{Att}}+k_{\text{Lo}}^{\text{Dis}}V_{\text{Lo}}^{\text{Att}}-\left(k_{\text{Hi}}^{\text{Att}}B_{\text{Hi}}+k_{\text{Lo}}^{\text{Att}}B_{\text{Lo}}\right)V^{\text{Ex}},$ (1)

$\text{with }B_{\text{n}}=B_{\text{n}}^{\text{tot}}-V_{\text{n}}^{\text{Att}}\text{ and }k_{\text{n}}^{\text{Dis}}=\frac{k_{\text{n}}^{\text{Att}}}{k_{\text{n}}^{\text{Eq}}} , n\in\left\{\text{Hi,Lo}\right\}.$ (2), (3)

Attached virions $V_{n}^{Att}$ either dissociate from binding sites with rate $k_{n}^{Dis}$ or are taken up by the cell via endocytosis with rate $k_{En}$. Then, virions in endosomes $V^{En}$ can fuse with the endosomal membrane and the viral genome is released to the cytoplasm $V^{Cyt}$, or, virions in endosomes are degraded with rate $k_{En}^{Deg}$.

$\frac{\text{d}V_{\text{n}}^{\text{Att}}}{\text{d}t}=k_{\text{n}}^{\text{Att}}B_{\text{n}}V^{\text{Ex}}-\left(k_{\text{n}}^{\text{Dis}}+k^{\text{En}}\right)V_{\text{n}}^{\text{Att}}, n\in\left\{\text{Hi,Lo}\right\},$ (4)

$\frac{\text{d}V^{\text{En}}}{\text{d}t}=k^{\text{En}}\left(V_{\text{Hi}}^{\text{Att}}+V_{\text{Lo}}^{\text{Att}}\right)-\left(k^{\text{Fus}}+k_{\text{En}}^{\text{Deg}}\right)V^{\text{En}},$ (5)

$\text{with }k_{\text{En}}^{\text{Deg}}=\frac{1-F_{\text{Fus}}}{F_{\text{Fus}}}k^{\text{Fus}}, 0<F_{Fus}\leq1,$ (6)

$\frac{\text{d}V^{\text{cyt}}}{\text{d}t}=8k^{\text{Fus}}V^{\text{En}}-k^{\text{Imp}}V^{\text{cyt}}.$ (7)

Viral RNA synthesis

Cytoplasmic vRNPs are imported into the nucleus with rate $k^{Imp}$, while newly synthesized vRNA $R^{V}$ is encapsidated, first, by binding of the viral polymerase with rate $k_{RdRp}^{Bind}$ and, secondly, binding of multiple copies of NP with rate $k_{NP}^{Bind}$. Furthermore, vRNPs bound to M1 $Vp_{M1}^{nuc}$ are exported with rate $k^{Exp}$ upon further binding of NEP.

$\frac{\text{d}Vp^{nuc}}{\text{d}t}=k^{\text{Imp}}V^{\text{Cyt}}+k_{\text{NP}}^{\text{Bind}}P_{\text{NP}}R_{\text{RdRp}}^{\text{V}}-\left(k_{\text{M1}}^{\text{Bind}}P_{\text{M1}}+k_{\text{Rnp}}^{\text{Deg}}\right)Vp^{nuc},$ (8)

$\frac{\text{d}R^{V}}{\text{d}t}=k_{\text{V}}^{\text{Syn}}Cp-k_{\text{RdRp}}^{\text{Bind}}P_{\text{RdRp}}R^{V}-k_{\text{R}}^{\text{Deg}}R^{V},$ (9)

$\frac{\text{d}R_{\text{RdRp}}^{\text{V}}}{\text{d}t}=k_{\text{RdRp}}^{\text{Bind}}P_{\text{RdRp}}R^{V}-k_{\text{NP}}^{\text{Bind}}P_{\text{NP}}R_{\text{RdRp}}^{\text{V}}-k_{\text{RRdRp}}^{\text{Deg}}R_{\text{RdRp}}^{\text{V}},$ (10)

$\frac{\text{d}Vp_{\text{M1}}^{\text{nuc}}}{\text{d}t}=k_{\text{M1}}^{\text{Bind}}P_{\text{M1}}Vp^{nuc}-\left(k^{\text{Exp}}P_{\text{NEP}}+k_{\text{Rnp}}^{\text{Deg}}\right)Vp_{\text{M1}}^{\text{nuc}}.$ (11)

During viral genome replication, viral cRNPs $Cp$ serve as replication intermediate that are synthesized from nuclear vRNPs with rate $k_{C}^{Syn}$ and are also encapsidated by the viral polymerase and NP molecules.

$\frac{\text{d}R^{C}}{\text{d}t}=k_{\text{C}}^{\text{Syn}}Vp^{nuc}-k_{\text{RdRp}}^{\text{Bind}}P_{\text{RdRp}}R^{C}-k_{\text{R}}^{\text{Deg}}R^{C},$ (12)

$\frac{\text{d}R_{\text{RdRp}}^{\text{C}}}{\text{d}t}=k_{\text{RdRp}}^{\text{Bind}}P_{\text{RdRp}}R^{C}-k_{\text{NP}}^{\text{Bind}}P_{\text{NP}}R_{\text{RdRp}}^{\text{C}}-k_{\text{RRdRp}}^{\text{Deg}}R_{\text{RdRp}}^{\text{C}},$ (13)

$\frac{\text{d}Cp}{\text{d}t}=k_{\text{NP}}^{\text{Bind}}P_{\text{NP}}R_{\text{RdRp}}^{\text{C}}-k_{\text{Rnp}}^{\text{Deg}}Cp.$ (14)

Viral mRNA and protein synthesis

Viral mRNA of each segment $i$ is synthesized from nuclear vRNPs with rate $k_{M}^{Syn}$.

$\frac{\text{d}R_{\text{i}}^{\text{M}}}{\text{d}t}=\frac{k_{\text{M}}^{\text{Syn}}}{L_{\text{i}}^{\text{M}}}\frac{Vp^{nuc}}{8}-k_{\text{M}}^{\text{Deg}}R_{\text{i}}^{\text{M}}, i=1,\ldots,8.$ (15)

The polymerase subunits PB2, PB1 and PA are translated from mRNAs of segments 1 to 3. They form the trimeric viral polymerase complex $P_{RdRp}$, that is attaching to naked vRNA and cRNA strands.

$\frac{\text{d}P_{\text{PB1}}}{\text{d}t}=\frac{k_{\text{P}}^{\text{Syn}}}{D_{\text{Rib}}}R_{2}^{\text{M}}-k^{\text{RdRp}}P_{\text{PB1}}P_{\text{PB2}}P_{\text{PA}},$ (16)

$\frac{\text{d}P_{\text{PB2}}}{\text{d}t}=\frac{k_{\text{P}}^{\text{Syn}}}{D_{\text{Rib}}}R_{1}^{\text{M}}-k^{\text{RdRp}}P_{\text{PB1}}P_{\text{PB2}}P_{\text{PA}},$ (17)

$\frac{\text{d}P_{\text{PA}}}{\text{d}t}=\frac{k_{\text{P}}^{\text{Syn}}}{D_{\text{Rib}}}R_{3}^{\text{M}}-k^{\text{RdRp}}P_{\text{PB1}}P_{\text{PB2}}P_{\text{PA}},$ (18)

$\frac{\text{d}P_{\text{RdRp}}}{\text{d}t}=k^{\text{RdRp}}P_{\text{PB1}}P_{\text{PB2}}P_{\text{PA}}-k_{\text{RdRp}}^{\text{Bind}}P_{\text{RdRp}}\left(R^{V}+R^{C}\right)-\left(N_{P_{RdRp}}-8\right)r^{Rel}.$ (19)

Furthermore, NP proteins, encoded by segment 5, are required to form replication-competent RNPs, which either participate in replication and transcription, or are exported from the nucleus upon binding of M1 and NEP, encoded by segment 7 and segment 8, respectively.

$\frac{\text{d}P_{\text{NP}}}{\text{d}t}=\frac{k_{\text{P}}^{\text{Syn}}}{D_{\text{Rib}}}R_{5}^{\text{M}}-\frac{L^{V}}{N_{\text{NP}}^{\text{Nuc}}}k_{\text{NP}}^{\text{Bind}}P_{\text{NP}}\left(R_{\text{RdRp}}^{\text{V}}+R_{\text{RdRp}}^{\text{C}}\right)-\left(N_{P_{NP}}-8\frac{L^{V}}{N_{\text{NP}}^{\text{Nuc}}}\right)r^{Rel},$ (20)

$\frac{\text{d}P_{\text{M1}}}{\text{d}t}=\frac{k_{\text{P}}^{\text{Syn}}}{D_{R\text{ib}}}\left(1-F_{\text{Spl7}}\right)R_{7}^{\text{M}}-\frac{L^{V}}{N_{\text{M1}}^{\text{Nuc}}}k_{\text{M1}}^{\text{Bind}}P_{\text{M1}}Vp^{nuc}-\left(N_{P_{M1}}-8\frac{L_{\text{m}}^{\text{V}}}{N_{\text{M1}}^{\text{Nuc}}}\right)r^{Rel},$ (21)

$\frac{\text{d}P_{\text{NEP}}}{\text{d}t}=\frac{k_{\text{P}}^{\text{Syn}}}{D_{\text{Rib}}}F_{\text{Spl8}}R_{8}^{\text{M}}-k^{\text{Exp}}P_{\text{NEP}}Vp_{\text{M1}}^{\text{nuc}}-\left(N_{P_{NEP}}-8\right)r^{Rel}.$ (22)

The viral surface proteins HA, NA and M2, which are encoded by segment 4, segment 6 and segment 7, respectively, are required for the formation of progeny virus particles and leave the cell during budding. For M2, the splicing of segment 7 is accounted for by including the fraction of spliced M2-mRNA $F_{Spl7}$. The M1 and other viral proteins are also incorporated into budding virions and leave the cell with release rate $r^{Rel}$.

$\frac{\text{d}P_{\text{HA}}}{\text{d}t}=\frac{k_{\text{P}}^{\text{Syn}}}{D_{\text{Rib}}}R_{4}^{\text{M}}-N_{\text{P}_{\text{HA}}}r^{\text{Rel}},$ (23)

$\frac{\text{d}P_{\text{NA}}}{\text{d}t}=\frac{k_{\text{P}}^{\text{Syn}}}{D_{\text{Rib}}}R_{6}^{\text{M}}-N_{\text{P}_{\text{NA}}}r^{\text{Rel}},$ (24)

$\frac{\text{d}P_{\text{M2}}}{\text{d}t}=\frac{k_{\text{P}}^{\text{Syn}}}{D_{\text{Rib}}}F_{\text{Spl7}}R_{7}^{\text{M}}-N_{\text{P}_{\text{M2}}}r^{\text{Rel}}.$ (25)

Assembly and release of virions

After nuclear export, progeny vRNPs in the cytoplasm $Vp_{M1}^{cyt}$ and viral proteins $P_{j}$ are assembling at the cellular plasma membrane. The number of released virions $V^{Rel}$ is directly depending on the number of available $Vp_{M1}^{cyt}$ and $P_{j}$ that influence virus release in a Michaelis-Menten-like fashion.

$\frac{\text{d}Vp_{\text{M1}}^{\text{cyt}}}{\text{d}t}=k^{\text{Exp}}P_{\text{NEP}}Vp_{\text{M1}}^{\text{nuc}}-8r^{Rel}-k_{\text{Rnp}}^{\text{Deg}}Vp_{\text{M1}}^{\text{cyt}}$ (26)

$\frac{\text{d}V^{\text{Rel}}}{\text{d}t}=r^{\text{Rel}}=k^{\text{Rel}}Vp_{M1}^{\text{cyt}}\prod_{j} \frac{P_{j}}{P_{j}+K_{V^{Rel}}N_{P_{j}}},$ (27)

$$\text{with }j\in\left\{\text{RdRp,HA,NA,NP,M1,M2,NEP}\right\}.$$

Supporting information references

1. Heldt FS, Frensing T, Reichl U. Modeling the Intracellular Dynamics of Influenza Virus Replication To Understand the Control of Viral RNA Synthesis. Journal of Virology. 2012. pp. 7806–7817. doi:10.1128/JVI.00080-12
